# *Bacillus cereus* T146 Enhances Wheat Salt Tolerance by Restructuring the Rhizosphere Microbiome and Activating TaPIN1‐Dependent Auxin Transport

**DOI:** 10.1111/pce.70567

**Published:** 2026-04-28

**Authors:** Fuqiang Zhu, Taotao Wang, Zhen Wang, Yelin Shan, Panrong Ren, Xiaomin Bie, Duojia Wang, Zheng Gao, Qingjie Guan, Lei Ge, Yuan Chen

**Affiliations:** ^1^ State Key Laboratory of Wheat Improvement, Peking University Institute of Advanced Agricultural Sciences Shandong Laboratory of Advanced Agricultural Sciences Weifang Shandong China; ^2^ National Key Laboratory of Wheat Improvement, College of Life Sciences Shandong Agricultural University Tai'an Shandong China; ^3^ School of Agriculture and Bioengineering Longdong University Qingyang Gansu China; ^4^ College of Life Sciences Northeast Agricultural University Harbin Heilongjiang China; ^5^ Key Laboratory of Saline-alkali Vegetation Ecology Restoration, Ministry of Education Northeast Forestry University Harbin Heilongjiang China; ^6^ The Characteristic Laboratory of Crop Germplasm Innovation and Application, Provincial Department of Education, College of Agronomy Qingdao Agricultural University Qingdao Shandong China; ^7^ Academician Workstation of Agricultural High-tech Industrial Area of the Yellow River Delta National Center of Technology Innovation for Comprehensive Utilization of Saline‐Alkali Land Dongying Shandong China

**Keywords:** auxin transport, *Bacillus cereus* T146, plant–microbe interaction, root development, salt tolerance, wheat

## Abstract

Salinity stress disrupts rhizosphere homoeostasis and inhibits root development. Although PGPR are known to alleviate such stress, critical knowledge gaps remain regarding the specific mechanisms by which they enhance tolerance under moderate to high salinity, particularly within the wheat rhizosphere ‐root interface. Here, we show that *Bacillus cereus* T146, isolated from saline‐alkali soil, enhances wheat salt tolerance through two integrated mechanisms. Metagenomic and culturomic analyses further revealed that T146 enriches IAA‐producing Pseudomonas in the rhizosphere, and co‐inoculation experiments demonstrated that these recruited bacteria contribute synergistically to salt tolerance. On the host side, transcriptomic and cell biological analyses demonstrated that T146 reactivates salt‐suppressed auxin pathways. Specifically, inoculation upregulates key regulators of lateral root development (*PLT3*, *PLT7*, *GLV6*) and increases *PIN1*, *PIN2*, and *PIN3* abundance, leading to elevated auxin accumulation as indicated by DR5::GFP signals. Importantly, silencing *TaPIN1* largely compromised T146‐induced tolerance and transcriptional reprogramming, demonstrating a functional interplay between microbiome modulation and host hormonal regulation. These results reveal that T146 synergistically promotes salinity resilience by coordinating rhizosphere microbiome remodelling with auxin‐mediated root development, offering a mechanistic framework for microbiome‐based strategies to improve crop stress tolerance.

## Summary statement

• *Bacillus cereus* T146 improves wheat salinity resilience through a dual mechanism: enriching IAA‐producing *Pseudomonas* in the rhizosphere and restoring host root auxin distribution via *TaPIN1*‐mediated transport.

## 1 Introduction

Soil salinization is a global agricultural and environmental challenge that severely restricts crop growth and productivity (Hassani et al. 2021; Mukhopadhyay et al. 2021). Driven by factors such as improper irrigation, rising sea levels, and climate change, over 1 billion hectares of land worldwide are affected by salinization, with the affected area continuing to expand (Eswar et al. 2021). Under high‐salt conditions, plants suffer from multiple stresses, including ion toxicity, osmotic stress, nutrient imbalance, and oxidative damage, leading to stunted growth, developmental abnormalities, and significant agricultural losses that threaten global food security (Tibesigwa et al. 2025; Van Zelm et al. 2020). Consequently, developing cost‐effective, efficient, and environmentally friendly strategies to enhance plant salt tolerance has become a critical priority for sustainable agriculture.

Plants do not passively endure environmental stress but have evolved sophisticated physiological and molecular mechanisms to cope with adversity (Zhang et al. 2022). Phytohormones function as key signalling molecules that integrate and coordinate adaptive responses to environmental stress (Waadt et al. 2022). As one of the earliest discovered and most pivotal plant hormones, auxin is not only essential for regulating growth and development but has also been identified as a key signalling molecule in plant responses to abiotic stresses, such as salt stress (Jing et al. 2023; Salehin 2024; Yu et al. 2020).

Under salt stress, auxin modulates root architecture, notably by promoting lateral root and root hair formation, maintains reactive oxygen species (ROS) homoeostasis, and regulates ion transport, particularly Na⁺/K⁺ balance, by redistributing within tissues and regulating downstream gene expression in key families including *AUX/IAA*, *GH3*, and *SAUR* (Ribba et al. 2020). These adaptations enhance water uptake, reduce toxic ion accumulation, and mitigate oxidative damage. However, prolonged or intense salt stress disrupts endogenous auxin synthesis and transport, impairing the plant's defence system. Thus, exogenous manipulation of auxin levels is considered a promising approach to bolster plant salt tolerance (Vanneste et al. 2025).

The plant root system harbours a complex and dynamic microbial community known as the rhizosphere microbiome (Bai et al. 2022; Pantigoso et al. 2022). While these microbial assemblies offer diverse benefits, their role in mediating salt stress tolerance via hormonal modulation is particularly critical. Among the various adaptive strategies employed by Plant Growth‐Promoting Rhizobacteria (PGPR), the capacity to synthesise and regulate Indole‐3‐Acetic Acid (IAA) stands out as a key determinant of root plasticity under saline conditions (Etesami and Glick 2024). Conventionally, the mechanism of PGPR‐mediated salt tolerance has been viewed through a linear lens: specific strains (e.g., *Bacillus*, *Pseudomonas*) are thought to directly secrete IAA, which is then passively absorbed by the host to optimise root architecture (Etesami and Glick 2020). However, this simplified model fails to capture the systemic complexity of the rhizosphere. It remains unclear whether keystone PGPR strains function solely as direct auxin providers or if they act as ecological hubs to recruit a broader consortium of auxin‐producing bacteria, thereby amplifying the adaptive signal (Berendsen et al. 2018; Trivedi et al. 2020). Furthermore, the specific host genetic interface that perceives and integrates these microbial auxin signals is poorly defined. Specifically, while studies in model plants suggest a role for auxin efflux carriers, the molecular link between microbiome‐mediated auxin enrichment and the host's endogenous auxin transport machinery (e.g., the PIN‐FORMED family) has not been causally established in major crops like wheat (Zamioudis et al. 2013). Addressing this disconnect between microbiome community assembly and host molecular signalling is essential for understanding plant resilience in high‐salinity environments. While PGPR has the potential to alleviate salt stress, a key question persists: how do salt‐tolerant, auxin‐producing strains coordinately remodel the rhizosphere microbiome and reprogramme host auxin signalling to mitigate salt stress.

In this study, we isolated a salt‐tolerant PGPR strain, *Bacillus cereus* T146, and investigated how it coordinates auxin‐related regulatory processes with rhizosphere microbial community assembly under salt stress. By integrating physiological, transcriptomic, and microbiome analyses, our study provides mechanistic insight into how beneficial microbes interact with host regulatory pathways during salt stress and offers a basis for understanding microbe‐assisted improvement of crop performance in saline environments.

## 2 Materials and Methods

### 2.1 Growth Tests of Strain T146 for Nitrogen Fixation, Phosphate Solubilisation, Siderophore Production, Potassium Release, and Salt Tolerance

For nitrogen fixation, phosphate solubilisation, siderophore production, and potassium release assays, bacterial suspensions were inoculated at four equidistant spots (5.0 μL per spot) on Ashby's nitrogen‐free medium, phosphate‐solubilizing medium, CAS agar, and potassium‐releasing solid medium, respectively. Each treatment was performed in triplicate, with plates labelled and incubated upside‐down at 28°C for 1 week. Growth was observed on each medium. To assess salt tolerance, a single colony of T146 was inoculated into TSB liquid medium and cultured overnight at 28°C with 200 rpm shaking. Then, 200 μL of TSB medium (with or without 500 mM NaCl for salt‐treated and control groups, respectively) was dispensed into a 96‐well plate. Bacterial suspension (2 μL) was added to each well, followed by incubation on a shaker (28°C, 200 rpm). To evaluate salt stress tolerance, the OD_600_ values were measured at 8‐h intervals over a 48‐h period using a microplate reader (Spark, Tecan).

### 2.2 Determination of Bacterial IAA Production Capacity

For the qualitative Analysis of IAA Production, the test strain was inoculated into R2A liquid medium supplemented with 1.0 g/L L‐tryptophan, cultured with shaking (180 rpm, 28°C) for 4 days. Fifty microliters of culture supernatant was mixed with an equal volume of Salkowski reagent (1 mL 0.5 mol/L FeCl₃ + 50 mL 35% perchloric acid) on a white ceramic plate, with 50 mg/L IAA standard as the positive control. The plate was incubated in the dark at 25°C for 30 min; a red colour indicated IAA synthesis capacity.

In order to quantitatively determine IAA concentration, first a standard curve needs to be prepared by dissolving 10 mg IAA in a small amount of absolute ethanol, diluting to 100 mL with distilled water to make a 100 μg/mL stock solution, preparing serial dilutions (0, 0.5, 1, 5, 10, 15, 20 μg/mL), mixing 5 mL of each standard with 5 mL Salkowski reagent, incubating in the dark at 25°C for 30 min, measuring absorbance at 530 nm (OD₅₃₀) using a UV‐visible spectrophotometer, and plotting a standard curve with IAA concentration (*y*‐axis) and OD₅₃₀ (*x*‐axis); bacterial cultures are then centrifuged at 10,000 rpm for 10 min to collect supernatants, 2 mL of which is mixed with 2 mL Salkowski reagent, reacted in the dark for 30 min, and OD₅₃₀ is measured to calculate IAA concentration in the fermentation broth via the standard curve.

### 2.3 Experiment on Testing Wheat Salt Tolerance With T146 Treatment

The wheat (Fielder) seeds were surface‐sterilised with 2% sodium hypochlorite for 10 min, rinsed three times with sterile water, and stratified in darkness at 4°C for 48 h to break dormancy. Sterilised seeds were sown in vermiculite and germinated for 5 days. To verify T146's salt tolerance‐enhancing and growth‐promoting effects on wheat, as well as its functional dependence on the native soil microbiome, seedlings of uniform growth were selected and transplanted into pots (9 × 9 cm) containing either sterilised or non‐sterilised soil (Sterilised soil: autoclaved at 121°C for 20 min (moist heat sterilisation); Non‐sterilised soil: Untreated soil). For each soil type, three treatments were applied: CK (1/2 Hoagland solution), Salt (1/2 Hoagland + 400 mM NaCl), and Salt + T146 (1/2 Hoagland + 400 mM NaCl + T146). Under conditions where interference from indigenous soil microorganisms was eliminated, to further evaluate the independent and synergistic effects of T146 and its recruited bacteria on wheat salt tolerance, a *Pseudomonas* strain (TN12) was isolated from salt‐stressed soil previously treated with T146 and selected as an auxiliary bacterium, uniformly grown seedlings were additionally transplanted into 9 × 9 cm pots filled with sterilised soil (moist heat sterilisation via autoclaving at 121°C for 20 min), with five treatments established: CK (1/2 Hoagland solution), NaCl (1/2 Hoagland solution + 400 mM NaCl), T146 (NaCl + T146), TN12 (NaCl + TN12), and MIX (NaCl + T146 + TN12).

To more intuitively observe T146's alleviating effect on wheat roots under salt stress, germinated seedlings were transplanted into 1 L hydroponic boxes and fixed with sterile sponges to ensure full root immersion. Four treatment groups were established (6 biological replicates per group, 2 seedlings per replicate): CK (1/2 Hoagland solution + 10% (v/v) non‐sterilised soil suspension), T146 (CK + T146), NaCl (CK + 200 mM NaCl), and NaCl+T146 (NaCl + T146).

For all inoculation treatments (including T146, TN12, and MIX), the bacterial inoculum concentration was uniformly adjusted to an OD₆₀₀ of 0.8. All plants were grown in a constant temperature incubator at 25°C under a 12 h light/12 h dark photoperiod for 2 weeks. Wheat growth was monitored, and physiological parameters including plant height, shoot fresh weight, tiller number, chlorophyll content, and lateral root density were measured.

In the subsequent functional validation experiments of transgenic wheat, *TaPIN1* overexpression (*TaPIN1‐*OE) and silencing (*TaPIN1*‐RNAi) lines were cultured in non‐sterilised soil under the same growth conditions and treatment regimes as the aforementioned soil‐based pot experiments, and the relevant physiological and growth parameters were recorded.

### 2.4 Metagenomic Sequencing

The samples used for metagenomic sequencing were the rhizosphere soil of wheat grown in non‐sterilised soil. Sampling was performed following the completion of the experiments for the CK, NaCl, and NaCl+T146 co‐treatment groups. A sterile brush was used to collect the rhizosphere soil into sterile centrifuge tubes. For each treatment group, rhizosphere soil from three randomly selected plants was pooled to form one composite sample. Three such composite samples were prepared per treatment group (*n* = 3 biological replicates). Each biological replicate therefore represents the pooled rhizosphere community from three individual plants, capturing within‐treatment variability while providing sufficient biomass for sequencing. The use of three independent composite samples per treatment allows statistical comparison of community composition among treatment groups. After collection, the samples were immediately flash‐frozen in liquid nitrogen and transferred to a −80°C ultra‐low temperature freezer for storage. First, soil DNA was extracted using the E.N.Z.A. Soil DNA Kit (Omega, USA). Subsequently, the soil DNA samples were sent to the company (Majorbio Bio‐Pharm Technology, Shanghai) for metagenomic sequencing. The raw sequencing data were quality‐controlled and filtered using fastp v0.23.0 by trimming adaptor sequences and removing low‐quality reads (length < 50 bp or quality value < 20), with an effective sequencing depth of 16 Gb clean data per sample. After assembly, contigs ≥ 300 bp were selected for gene prediction via Prodigal (ORFs ≥ 100 bp were retained). A non‐redundant gene catalogue was constructed using CD‐HIT v4.6.1 with 90% sequence identity and 90% coverage, and its representative sequences were aligned to the NR, eggNOG and KEGG databases for taxonomic and functional annotations using Diamond v0.8.35 (e‐value cutoff = 1e‐5). Raw gene abundance was calculated by aligning clean reads to the non‐redundant gene catalogue at 95% identity via SOAPaligner v2.21, and normalised by the TPM method to eliminate sequencing depth differences. For differential abundance analysis, a pseudo‐count of 1e‐6 was added to TPM values followed by CLR transformation to correct compositional bias. Based on phylum and genus‐level species abundance tables, R language was used for alpha diversity (Ace, Shannon, Simpson indices), beta diversity (PCoA_NR scatter plot), hierarchical clustering and multi‐species difference tests. Statistical significance was set at *p* < 0.05, with one‐way ANOVA followed by Tukey HSD post‐hoc test for ≥ 3 group comparisons and two‐tailed Student's *t*‐test for two‐group comparisons.

### 2.5 RNA‐Seq Library Preparation and Sequencing

The processed wheat root samples were collected and rinsed with purified water to remove impurities. Each treatment group included three biological replicates. A portion of the samples was stored in a −80°C ultra‐low temperature freezer, while the remaining samples were sent to Novogene Bioinformatics Technology (Tianjin, China) for library construction and sequencing. Raw reads were processed to remove adaptors, poly‐N sequences, and low‐quality reads. Quality metrics including Q20, Q30, and GC content were calculated for the clean data. All subsequent analyses were performed using this high‐quality processed dataset. Clean reads were aligned to the *Triticum aestivum* genome using HISAT2 (v2.0.5), and the FPKM value for each gene was quantified using Feature Counts (1.5.0‐p3). To ensure the reliability of the data, we removed the genes whose FPKM values were zero. For each gene, the average FPKM value across three biological replicates was calculated for each treatment group. Differential expression between comparison groups was determined based on the fold change of average FPKM values. Genes with an absolute log₂‐ transformed fold change (|log₂FC|) ≥ 1 were identified as differentially expressed genes (DEGs).

### 2.6 Enrichment Analysis of Differentially Expressed Genes

Gene Ontology (GO) enrichment analysis was performed using Agrigo (https://systemsbiology.cau.edu.cn/agriGOv2/). The annotation terms of biological process (BP), cellular component (CC), and molecular function (MF) were selected and visualised using ggplot2 in R‐studio. The KEGG (Kyoto Encyclopedia of Genes and Genomes) enrichment analysis was performed on the website (https://www.omicshare.com/tools/). All figures were assembled in Adobe Illustrator.

### 2.7 T146 Treatment and Phenotypic Observation of *Arabidopsis* Seedlings

*Arabidopsis thaliana* seeds were surface‐sterilised with 75% ethanol for 5 min, rinsed five times with ddH_2_O, and stratified at 4°C for 2 days before being plated on 1/2 Murashige and Skoog (1/2 MS) medium (Duchefa, Netherlands) containing 0.8% (w/v) agar, 1% (w/v) sucrose, pH 5.7. Plants were grown under a 16‐h light (22°C)/8‐h dark (18°C) photoperiod with a photosynthetic photon flux density of 120 µE m⁻² sec⁻¹ in a growth room.

To investigate the effect of T146 on the salt tolerance of *Arabidopsis thaliana*, 4‐day‐old wild‐type (ecotype Columbia‐0, Col‐0) seedlings grown on standard solid 1/2 MS medium were transferred to fresh medium under four conditions: control, T146‐supplemented, 100 mM NaCl‐supplemented, and NaCl+T146‐supplemented. The seedlings were cultivated for an additional 5 days, after which the primary root elongation length and the number of lateral roots were quantified. To examine whether the potential promotion of salt tolerance by T146 is mediated by auxin biosynthesis, 10 μM NPA (N‐1‐naphthylphthalamic acid) was added to the NaCl+T146‐supplemented 1/2 MS medium. The growth of wild‐type *Arabidopsis* primary roots was observed following transplantation.

To optimise the interaction efficiency of mixed bacteria (T146, TN12, and their mixture) and facilitate better observation of their effects, a hydroponic system was established for *Arabidopsis thaliana*. The system used water agar medium 0.6% (w/v) agar, where the aboveground parts of seedlings were placed on the agar surface, and roots were immersed in sterile 1/5 Hoagland solution. All hydroponic treatments were conducted with five groups designated: CK (1/5 Hoagland solution), NaCl (sterile 1/5 Hoagland solution + 50 mM NaCl), T146 (NaCl + T146), TN12 (NaCl + TN12), and MIX (NaCl + T146 + TN12). Seedlings were cultured under the aforementioned photoperiod and temperature conditions, and root growth parameters were measured to assess the independent and synergistic effects of the mixed bacteria.

### 2.8 Quantitative RT‐PCR (qRT‐PCR)

All RNA samples from *Arabidopsis* roots were extracted using the plant RNA isolation kit (R6731‐00S, Omega). 1 μg of total RNA was reverse transcribed by the HiScript III All‐in‐one RT SuperMix (R333‐01, Vazyme). qRT‐PCR was performed using ChamQ Universal SYBR qPCR Master Mix (Q711‐02, Vazyme) to assess the expression level of lateral root development associated genes on the Real‐Time PCR System (QuantStudio 5, Thermo Fisher). The relative expression of target genes was analyzed using the 2^−ΔΔCt^ method, and the expression of the control was normalised to 1. *UBC* was used as the internal control. Primers used are listed in Table S1.

### 2.9 Confocal Microscopy

To observe the expression of auxin transport‐related proteins following T146 and NaCl treatments, 4‐day‐old seedlings expressing PIN1‐GFP, PIN2‐GFP, PIN3‐GFP, and DR5‐GFP grown on standard 1/2 MS medium were transferred to fresh medium under four conditions: control, T146‐supplemented, NaCl‐supplemented, and NaCl+T146‐supplemented, followed by an additional 5 days of growth. All confocal images were captured using a Nikon B3 confocal microscope (Japan). GFP was excited at 488 nm, with emission collected at 500–550 nm.

### 2.10 Statistics Analysis

Primary root length and fluorescence intensity were measured using ImageJ. Statistical analyses were performed using SPSS (v26.0). For comparisons involving two groups, unpaired two‐tailed Student's *t*‐tests were used. For multi‐group comparisons (three or more groups), one‐way ANOVA followed by Tukey's HSD post‐hoc test was applied. Significance levels are indicated as *p < 0.05, **p < 0.01, ***p < 0.001, and ****p < 0.0001. Experiments were replicated at least three times, and representative data are shown. Bar charts and box plots were generated using GraphPad Prism 8, and figures were assembled in Adobe Illustrator CS6.

## 3 Results

### 3.1 *B. cereus* T146 Enhances Salt Tolerance in Wheat Under Non‐Sterilised Soil Conditions

Rhizosphere microorganisms are essential to improving plant salt tolerance. To identify rhizobacteria capable of enhancing plant salt resistance, we screened and isolated a salt‐tolerant strain from the saline–alkali rhizosphere of saline–alkali pioneer plants (*Suaeda salsa* and *Puccinellia tenuiflora*), which act as constructive species in saline grasslands and have evolved under extreme selection pressures that favour the recruitment of highly salt‐tolerant microbiome assemblages via specific metabolic cues (Li and Song 2019; Chen et al. 2022). 16S rRNA gene sequencing and phylogenetic analysis identified the strain as *Bacillus cereus* T146 (Figure S1A). In vitro assays revealed that T146 possesses multiple plant growth‐promoting properties, including the ability to solubilise phosphate (P), produce siderophores [CAS(Fe)], and synthesise indole‐3‐acetic acid (IAA) (Figure S1B,C). In addition, the strain maintained significant growth capacity under 500 mM NaCl.

**Figure 1:**
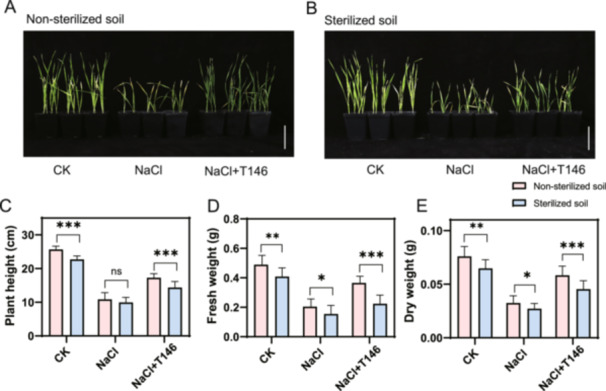
*B. cereus* T146 enhances salt stress tolerance in wheat under different soil treatment conditions. (A) Phenotypic performance of Fielder wheat under different treatments in non‐sterilised soil. CK (control, no salt stress); NaCl (400 mM NaCl); NaCl+T146 (inoculation with *B. cereus* T146 combined with 400 mM NaCl). 5‐day‐old Fielder wheat plants with uniform growth were used. (B) Phenotypic performance of Fielder wheat under different treatments in sterilised soil. CK (control, no salt stress); NaCl (400 mM NaCl); NaCl+T146 (inoculation with *B. cereus* T146 combined with 400 mM NaCl). 5‐day‐old Fielder wheat plants with uniform growth were used. (C) Plant height of Fielder wheat under different treatments. (D) Fresh weight of Fielder wheat under different treatments. (E) Dry weight of Fielder wheat under different treatments. Scale bar = 10 cm. In (C), (D), and (E), data are presented as mean ± SD (*n* ≥ 15). Comparisons among three groups were performed by one‐way ANOVA, followed by Tukey HSD test. Different letters (a, b, c, d, e) indicate significant differences between groups (*p* < 0.05).

To evaluate the functional potential of T146 in crops, we investigated its effects on wheat growth under salt stress. Salt stress significantly reduced wheat biomass (including plant height, fresh weight, and dry weight). In non‐sterilised soil, inoculation with T146 (NaCl+T146 treatment) alleviated this salt‐induced inhibition: compared to the NaCl‐treated control, T146 inoculation significantly increased plant height, fresh weight, and dry weight (Figure 1A,C–E). Physiological analyses showed that salt stress markedly increased malondialdehyde (MDA) levels in wheat leaves, indicating enhanced membrane lipid peroxidation (Figure S1E), while activities of superoxide dismutase (SOD) and peroxidase (POD) were elevated (Figure S1F,G)—reflecting an adaptive oxidative stress response. In contrast, T146 inoculation significantly reduced MDA accumulation and restored SOD and POD activities to near‐control levels (Figure S1E–G), demonstrating that T146 mitigates salt‐induced oxidative damage by enhancing wheat's antioxidant capacity. Notably, inoculation with T146 also mitigated salt stress in sterilised soil, but the alleviating effect was less pronounced compared to that in non‐sterilised soil (Figure 1B–E). Specifically, in sterilised soil, the NaCl+T146 treatment resulted in modest improvements in wheat plant height, fresh weight, and dry weight relative to the NaCl‐treated control; however, these growth‐promoting increments were significantly smaller than those observed in non‐sterilised soil, indicating that T146‐mediated salt tolerance depends on synergistic interactions with the native soil microbiome.

### 3.2 Metagenomic Analysis Reveals T146 Inoculation Enriches Beneficial Microbes in the Wheat Rhizosphere

To elucidate the contribution of rhizosphere microbial communities to T146‐mediated salt tolerance and to evaluate the colonisation capacity of strain T146 in wheat, we combined metagenomic sequencing with culturomic isolation of rhizosphere samples. Metagenomic analysis revealed a significant enrichment of *B. cereus* in the T146‐inoculated group (NaCl+T146) relative to both the NaCl‐only and CK controls (Figure S2A). Consistent with this result, isolation of bacteria from NaCl+T146 rhizosphere soil using a selective medium identified one strain, designated TN35, whose 16S rRNA gene sequence clustered with the T146 reference strain and showed 100% sequence identity (Figure S2B). These findings demonstrate that strain T146 survives and stably colonises the wheat rhizosphere under salt stress.

**Figure 2:**
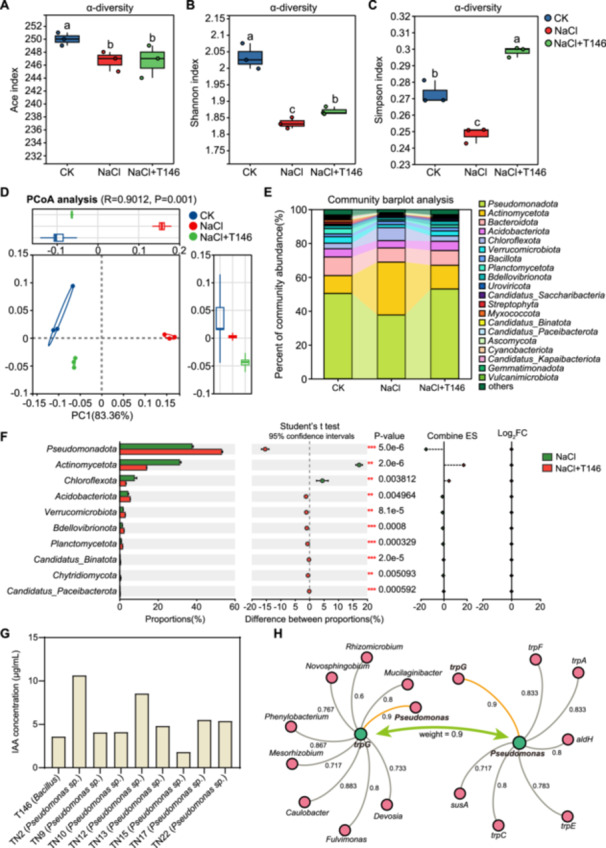
Metagenomic analysis of the rhizosphere microbial community in response to salt stress and T146 inoculation. (A–C) Alpha diversity indices of the wheat rhizosphere microbial community under different treatments, including (A) Ace index, (B) Shannon index, and (C) Simpson index. Data are presented as mean ± SD (*n* = 3). Comparisons among three groups were performed by one‐way ANOVA, followed by Tukey HSD test. Different letters (a, b, c) indicate significant differences between groups (*p* < 0.05). CK (control, no salt stress, no inoculation), NaCl (400 mM NaCl), NaCl+T146 (inoculation with strain T146 + 400 mM NaCl). Rhizosphere soil samples for sequencing were collected two weeks after treatment. (D) Principal coordinate analysis (PCoA) based on Bray–Curtis distances, showing beta diversity of microbial communities among different treatment groups. Percentages indicate the variance explained by PC1 and PC2. The ANOSIM result (*R* = 0.9012, *p* = 0.001) confirms significant separation between groups. (E) Community bar plot showing the relative abundance of microbial taxa at the phylum level under each treatment. (F) Differences in the average relative abundance at the phylum level between the NaCl and NaCl+T146 groups (Significance notation: **p ≤ 0.01, ***p ≤ 0.001, Student's *t*‐test). (G) Determination of IAA Production by Strain T146 and other *Pseudomonas* Strains. (H) Correlation network analysis of connection coefficients between beneficial rhizosphere bacteria recruited by T146 (e.g., *Pseudomonas* sp.) and key functional genes (e.g., *trpC, trpE*) (Correlations via R package WGCNA, Spearman's r > 0.7, *p* < 0.05). [Color figure can be viewed at wileyonlinelibrary.com]

Beyond confirming T146 establishment, rhizosphere metagenomic profiling further revealed substantial alterations in microbial community structure under saline conditions. In particular, NaCl treatment markedly reduced microbial α‐diversity compared with the control (CK), as indicated by significant declines in the Simpson, Shannon, and Ace indices (Figure 2A–C), providing a community‐level context for subsequent analyses of T146‐mediated microbiome modulation. Inoculation with T146 partially alleviated these effects, showing a clear recovery of the Simpson index and adaptive adjustment trends in the Ace and Shannon indices. Collectively, these results indicate that T146 contributes to the restoration of rhizosphere microbial diversity under saline conditions.

Principal coordinate analysis (PCoA) revealed clear separation among different treatment groups (PC1 explained 83.36% of the variance) (Figure 2D). The NaCl‐treated group differed significantly from both the control (CK) and NaCl+T146 groups, indicating that salt stress markedly altered microbial community composition, while T146 inoculation partially restored the community structure, shifting it away from the salt‐stressed state (Figure 2D). Moreover, hierarchical clustering analysis confirmed good reproducibility among the biological replicates (Figure S2C). These results suggest that both NaCl treatment and NaCl+T146 treatment exert a strong selective effect on the soil microbial community, and that T146 addition under salt stress helps promote recovery of the rhizosphere microbiome.

To further investigate how T146 shapes the rhizosphere microbial community under salt stress, we analyzed the taxonomic composition and relative abundances of microbial taxa. Salt stress (NaCl treatment) significantly altered the soil microbial community structure, with notable shifts in several dominant phyla (Figure 2E, Figure S2D). In the NaCl‐treated group, the relative abundance of *Pseudomonadota*, a key phylum involved in nutrient cycling and plant growth promotion, decreased significantly compared with the control (CK) (*p* < 0.05), while *Acidobacteriota* and *Chloroflexota* showed slight increases (Figure 2F). In contrast, inoculation with T146 *(Bacillus* sp.) under NaCl stress induced a pronounced restorative effect on the microbial community. The relative abundance of *Pseudomonadota* significantly recovered compared with the NaCl group (*p* < 0.05) and regained its status as a dominant phylum, approaching levels observed in the CK group. Other dominant phyla, including *Actinomycetota*, *Chloroflexota*, *Acidobacteriota*, and *Verrucomicrobiota*, also exhibited significant recovery toward CK levels under T146 treatment (*p* < 0.05) (Figure 2F). To clarify the recruitment effect of T146 on functional microbial taxa under salt stress, we conducted isolation and functional characterisation analyses targeting rhizosphere soil from the NaCl+T146 treatment (i.e., rhizosphere soil under NaCl stress with T146 inoculation). To this end, we used a tryptophan‐containing selective medium for bacterial screening and subsequently successfully isolated eight distinct *Pseudomonas strains* (TN2, TN9, TN10, TN12, TN13, TN15, TN17, and TN22). All eight strains were capable of synthesising indole‐3‐acetic acid (IAA) (Figure 2G). We further explored the functional associations between the recruited microbial community and key functional genes based on KEGG Orthology annotations, using correlation‐weight analysis (Figure 2H). The results showed that genes closely related to tryptophan biosynthesis (*trpA, trpC, trpF, trpE*, and *trpG*) and genes involved in carbon metabolism (*aldH, susA*) exhibited strong associations (*R* > 0.7) with functional genera including *Rhizomicrobium*, *Novosphingobium*, and *Pseudomonas*. Notably, *Pseudomonas* showed the highest association with the tryptophan biosynthesis gene *trpG* (*R* = 0.9), confirming its role as a core functional genus involved in IAA production under salt stress.

Given that tryptophan is a key precursor for IAA biosynthesis, these results, together with the strain isolation data and gene association analysis, suggest that T146 enhances the rhizosphere environment under salt stress both by directly producing IAA and by reshaping the microbial community to recruit functional taxa, particularly *Pseudomonas*, which promotes tryptophan biosynthesis and indirectly regulates IAA metabolism to alleviate salt‐induced growth inhibition.

### 3.3 Transcriptomic Profiling Reveals T146 Induces Auxin Signalling Responses in Wheat Roots

**Figure 3:**
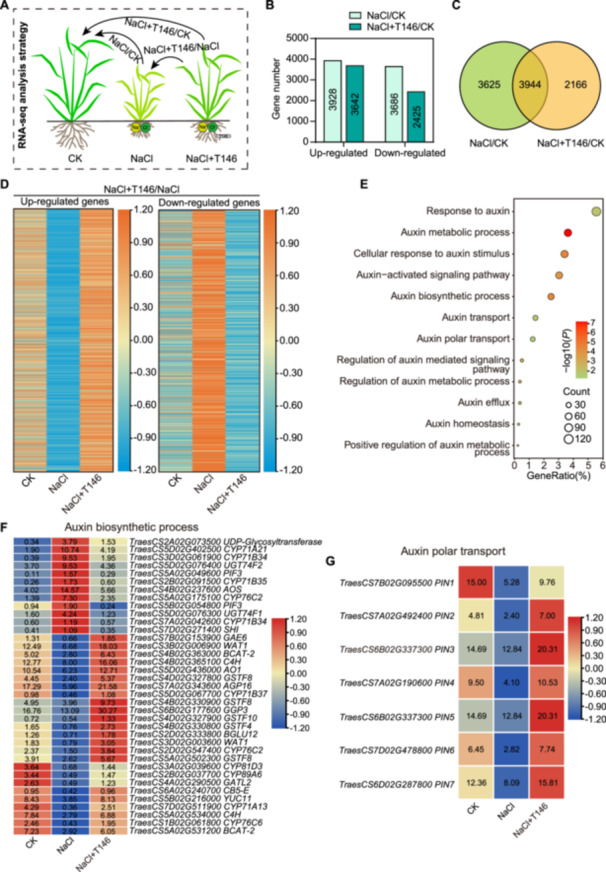
Transcriptomic profiling of wheat roots reveals T146‐mediated reprogramming of gene expression under salt stress. (A) Schematic of RNA‐seq experimental design and alignment. Fielder wheat plants were treated as follows: CK (control), NaCl (400 mM NaCl), and NaCl+T146 (inoculation with T146 plus 400 mM NaCl). Roots were harvested for RNA‐seq analysis. (B) Bar graph displaying the number of differentially expressed genes (DEGs) (|log₂FC| > 1) in pairwise comparisons between NaCl/CK and NaCl+T146/CK. Up‐regulated and down‐regulated genes are shown. (C) Venn diagram showing overlapping DEGs between the NaCl/CK and NaCl+T146/CK comparisons. (D) Heatmap of gene expression profiles (Z‐scores) for DEGs specifically identified in the NaCl+T146 versus NaCl comparison. (E) Gene Ontology (GO) enrichment analysis of biological processes related to auxin in the comparison between NaCl+T146 and NaCl treatments. The colour scale represents the statistical significance (‐log₁₀(*p*‐value)), and the dot size indicates the number of genes in each term. (F) Expression patterns of key auxin biosynthetic genes under different treatments (CK, NaCl, NaCl+T146). The heatmap displays log₂‐ transformed fold changes, illustrating that salt stress (NaCl) suppresses, while T146 application (NaCl+T146) restores, the expression of these critical auxin pathway genes. (G) Expression patterns of key polar transport genes under CK, NaCl and NaCl+T146 treatment. [Color figure can be viewed at wileyonlinelibrary.com]

To dissect the molecular mechanisms underlying T146‐mediated salt tolerance in wheat, we conducted RNA‐seq analysis on roots of wheat seedlings exposed to three treatment conditions: optimal growth conditions (no salt stress, no T146 inoculation), salt stress alone, and the combination of salt stress and T146 inoculation (Figure 3A). Differential gene expression analysis showed that salt stress (NaCl treatment) resulted in 7,570 differentially expressed genes (DEGs) compared to the control (CK), with 3,928 up‐regulated and 3,642 down‐regulated. In contrast, the T146‐inoculated salt‐stressed group (NaCl+T146) exhibited only 6,111 DEGs (3,686 up‐regulated and 2,425 down‐regulated) compared to CK (Figure 3B), indicating that T146 inoculation reduced the transcriptional disruption caused by salt stress. Among these, 3,945 genes were commonly differentially expressed in both NaCl and NaCl+T146 treatments (Figure 3C). Further comparison between the NaCl+T146 and NaCl groups revealed that T146 inoculation reversed the expression patterns of a majority of salt‐responsive genes based on differential expression analysis (Figure 3D), suggesting that T146 may enhance salt tolerance by modulating key signalling pathways. GO enrichment analysis indicated that T146 significantly altered biological processes such as cell wall formation, biomass regulation, and DNA replication (Figure S3A). Notably, multiple auxin‐related processes were affected by T146 treatment, including auxin efflux, polar auxin transport, auxin biosynthetic and metabolic process, auxin‐activated signalling pathway, and cellular response to auxin stimulus (Figure 3E).

We further analyzed the expression of specific genes. As shown in Figure 3F, salt stress significantly suppressed the expression of several key auxin biosynthetic genes (such as *YUC11*, *CYP71A13*, and *CYP76C6*). In contrast, T146 treatment markedly up‐regulated the expression of these genes (Figure 3F). This suggests that T146 may enhance the endogenous auxin levels under salt stress by activating the auxin biosynthesis pathway, thereby promoting salt tolerance. Similarly, the expression of PIN family genes (such as *PIN1*, *PIN2*, and *PIN3*), which encode auxin efflux carriers, was generally down‐regulated under salt stress, while T146 application largely restored their expression (Figure 3G), indicating that T146 may help maintain proper auxin distribution within plant tissues. KEGG pathway analysis further demonstrated that T146 inoculation significantly influenced several metabolic pathways, including amino sugar and nucleotide sugar metabolism, phenylpropanoid biosynthesis, glycolysis/gluconeogenesis, and starch and sucrose metabolism (Figure S3B). Additionally, pathways related to stress response and redox homoeostasis—such as peroxidase activity, antioxidant activity, and glutathione metabolism—were notably enriched (Figure S3B). These findings are consistent with the possibility that T146 contributes to wheat adaptation to salt stress through enhanced antioxidant capacity and energy metabolism efficiency.

### 3.4 T146 Alleviates Salt‐Induced Inhibition of Root System Development in Wheat and *Arabidopsis*

**Figure 4:**
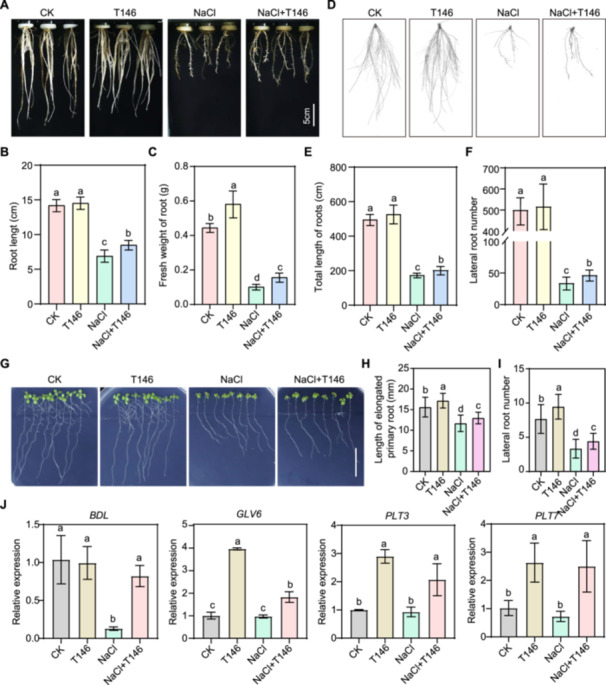
*B. cereus* T146 alleviates salt‐induced inhibition of root system development in wheat and *Arabidopsis*. (A) Phenotypic performance of Fielder wheat roots after different treatments in Hoagland nutrient solution. CK (control, no salt stress); T146 (inoculation with T146); NaCl (200 mM NaCl); NaCl+T146 (inoculation with strain T146 combined with 200 mM NaCl). 5‐day‐old Fielder wheat plants with uniform growth were used for treating. Non‐sterilised soil suspensions were added in equal amounts to different treatments respectively. Scale bar = 5 cm. (B) Statistics of primary root length of Fielder wheat after different treatments in (A). (C) Statistics of fresh weight of the Fielder wheat roots under different treatments in (A). (D) Root scan images of Fielder wheat after different treatments in (A). (E) Statistics of total length of the wheat roots based on the scanning result from (D). (F) Statistics of the lateral root number of wheat with different treatments based on the scanning result from (D). In (B), (C), (E), and (F), (G) Phenotype of *Arabidopsis* seedlings grown under CK, T146, NaCl and NaCl+T146 treatments. Four‐day‐old wild ‐type seedlings were transferred to 1/2 MS solid media with the following treatments: CK (control), T146 (inoculation with T146), NaCl (100 mM), and NaCl +T146 (inoculation with T146 plus 100 mM NaCl), and grown vertically for an additional 7 days. Scale bar = 1 cm. (H) Statistical analysis of elongated primary root length shown in (G). (I) Statistics of the lateral root number of *Arabidopsis* seedlings after different treatments in (G). (J) Quantitative PCR (qPCR) analysis of the relative expression levels of key lateral root development‐related genes (*BDL*, *GLV6*, *PLT3* and *PLT7*) in *Arabidopsis* roots under different treatments. Data are presented as mean ± SD (*n* ≥ 15; qPCR replicates = 3). Comparisons among four groups were performed by one‐way ANOVA, followed by Tukey HSD test. Different letters (a, b, c and d) indicate significant differences between groups (*p* < 0.05). [Color figure can be viewed at wileyonlinelibrary.com]

Transcriptome analysis indicated that T146 counteracts salt‐induced down‐regulation of key auxin biosynthesis and PIN transport genes, suggesting its role in maintaining auxin homoeostasis under stress (Figure 3F,G). In addition, T146 also enriches beneficial IAA‐producing microorganisms in the wheat rhizosphere (Figure 2). Given the well‐established role of auxin in modulating root architecture and stress responses (Jing et al. 2023), we hypothesised that T146 enhances plant salt tolerance by enhancing auxin‐mediated root development. To test this hypothesis, we first examined the effects of T146 on wheat root growth under salt stress. Phenotypic observations revealed that NaCl treatment markedly reduced primary root length and lateral root number compared with the control (Figure 4A–F). In contrast, T146 inoculation largely restored these traits, resulting in significantly longer roots, higher fresh biomass, and more lateral roots than in NaCl‐treated plants (Figure 4A–F), indicating that T146 effectively alleviates salt‐induced inhibition of root growth. To further examine how T146 influences auxin transport and signalling, we extended our analysis to *Arabidopsis thaliana*, a model system with well‐established genetic tools for studying auxin‐related pathways. In *Arabidopsis*, T146 inoculation significantly alleviated the salt‐induced inhibition of root system development, resulting in longer primary roots and an increased number of lateral roots compared with NaCl‐treated plants (Figure 4G–I).

This phenotypic observation was corroborated by the analysis of key genetic regulators of lateral root development. The expression of *PLT3* and *PLT7* (Figure 4J), which are crucial for root stem cell maintenance and lateral root initiation, was significantly down‐regulated by salt stress. T146 treatment effectively restored their expression to levels comparable to or even exceeding the control (CK) (Figure 4J). Similarly, the expression of *GLV6* (a peptide hormone gene known to promote cell division and root development) and *BDL* (an AUX/IAA transcriptional repressor that regulates primary root meristem formation) was strongly suppressed by salt stress but markedly induced by T146 (Figure 4J). These coordinated molecular regulatory changes indicate that T146 promotes lateral root development under salt stress likely by modulating the expression of key regulatory genes—specifically activating *PLT3*, *PLT7*, *GLV6* and *BDL*. To investigate whether the beneficial effect of T146 operates through the auxin pathway, we observed that T146 rescued salt‐inhibited root growth traits, including primary root length and lateral root number. Crucially, this protective effect was substantially negated by the auxin transport inhibitor NPA, with the T146+NaCl+NPA group displaying phenotypes comparable to the NaCl‐treated controls. This indicates that T146‐mediated tolerance is critically dependent on functional auxin transport. Beyond primary and lateral roots, we further investigated the response of root hairs (key for water and nutrient uptake). Salt stress significantly reduced root hair length compared to the CK (Figure S4E,F). Notably, T146 inoculation significantly promoted root hair growth under salt stress, as evidenced by the increased root hair length in the T146+NaCl group compared to the NaCl group (Figure S4E,F). These findings confirm that the ability of T146 to enhance salt tolerance by modulating root architecture is effective in plants.

### 3.5 T146 Upregulates Auxin Transporter Expression in Roots

**Figure 5:**
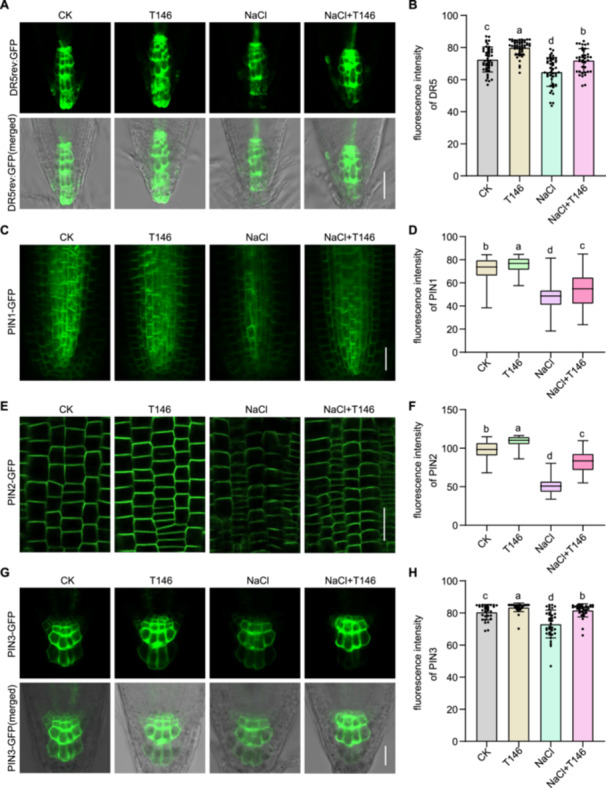
*B. cereus* T146 modulates auxin accumulation and polar transport by regulating PIN protein abundance in *Arabidopsis* roots. (A) Representative confocal microscopy images of root tips expressing DR5rev:GFP under different treatments (CK, T146, NaCl, NaCl+T146). 4‐day‐old seedlings were transferred and grown on fresh medium for 5 days before imaging. Bright‐field and merged channels are shown. Scale bar = 50 μm. (B) Quantification of *DR5rev:GFP* fluorescence intensity in the columella from (A). (C) Confocal images of root tips expressing PIN1‐GFP after treatments (CK, T146, NaCl, NaCl+T146). 4‐day‐old seedlings were transferred and grown for 5 days before imaging. Scale bar = 20 μm. (D) Quantitative analysis of PIN1‐GFP fluorescence intensity from (C). (E) Confocal images of root tips expressing PIN2‐GFP after treatments (CK, T146, NaCl, NaCl+T146). Processing conditions same as (C). Scale bar = 20 μm. (F) Quantitative analysis of PIN2‐GFP fluorescence intensity from (E). (G) Confocal images of root tips expressing PIN3‐GFP after treatments (CK, T146, NaCl, NaCl+T146). Processing conditions same as (C). Scale bar = 20 μm. (H) Quantitative analysis of PIN3‐GFP fluorescence intensity from (G). Data are presented as mean ± SD (*n* = 10). Comparisons among four groups were performed by one‐way ANOVA, followed by Tukey HSD test. Different letters (a–d) indicate significant differences between groups (*p* < 0.05). [Color figure can be viewed at wileyonlinelibrary.com]

In wheat, functional characterisation of auxin transporters was technically constrained, as attempts to visualise fluorescently tagged PIN proteins were unsuccessful. Therefore, we extended our analysis to *Arabidopsis thaliana*, a model system with well‐established genetic tools for studying auxin‐related pathways, to dissect the cellular mechanisms underlying T146's effects on auxin transport. To further dissect the auxin‐related mechanisms by which T146 enhances salt tolerance, we utilised auxin reporter lines and fluorescently tagged auxin transporters in *Arabidopsis* to examine its effects on auxin distribution and the expression of polar transport proteins in the root apex (Chen et al. 2013; Xiao et al. 2020). First, salt stress (NaCl) with a long term significantly reduced DR5rev::GFP signal intensity in the root tip, indicating decreased auxin accumulation (Figure 5A,B). Inoculation with T146 (NaCl+T146) markedly restored DR5 signal intensity (*p* < 0.001) (Figure 5A,B), demonstrating that T146 counteracts salt‐induced auxin depletion. When the auxin transport inhibitor NPA was applied simultaneously (T146+NaCl+NPA) (Abas et al. 2021), DR5 signal intensity was again suppressed (Figure S5A,B), confirming that T146's effect on auxin accumulation depends on functional polar auxin transport.

To further elucidate the molecular basis of T146‐regulated auxin transport, we examined the expression and localisation of several PIN auxin efflux carriers (Adamowski and Friml 2015; Křeček et al. 2009). Under salt stress, the fluorescence intensity of both PIN1, PIN2 and PIN3 protein was significantly reduced (Figure 5C–H). T146+NaCl treatment substantially up‐regulated their expression (Figure 5C–H), with the proteins maintaining plasma membrane localisation, indicating that T146 specifically enhances PIN1‐, PIN2 ‐ and PIN3‐ mediated auxin transport. No significant changes were observed in PIN7::GFP across treatments (Figure S5C,D), suggesting that T146's regulation of PIN proteins is member‐specific. These results indicate that T146 specifically upregulates the expression of auxin efflux carriers such as *PIN1*, *PIN2*, and *PIN3*, thereby enhancing polar auxin transport and to form better optimal concentration gradient in the root tip. This mechanism promotes primary root elongation and lateral root formation under salt stress.

### 3.6 Silencing *Tapin1* Impairs T146‐Induced Salt Tolerance in Wheat

**Figure 6:**
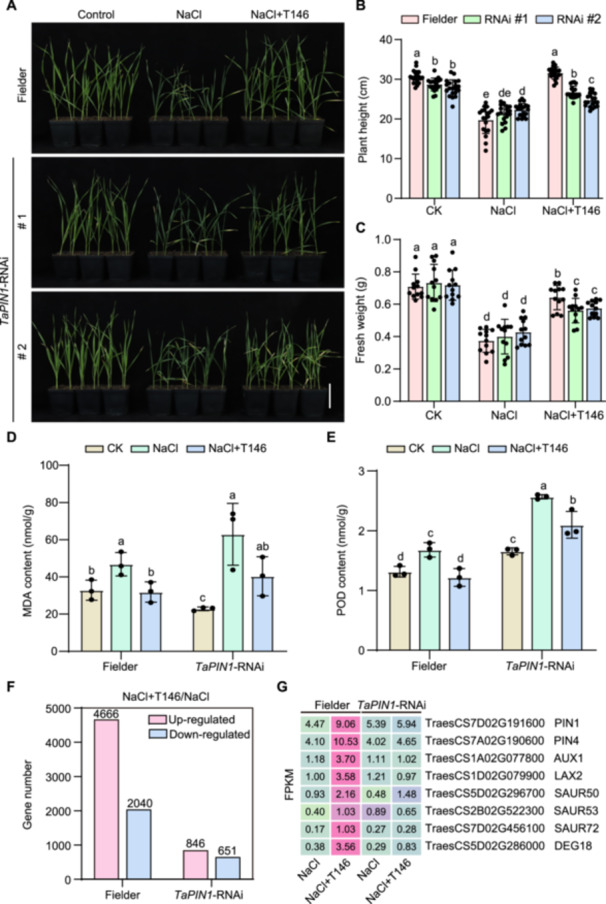
Silencing of *TaPIN1* abolishes the salt tolerance conferred by *B. cereus* T146 in wheat. (A) Representative images of wild‐type (Fielder) and *TaPIN1*‐RNAi wheat plants under different treatments: CK (control), NaCl (400 mM NaCl), and NaCl+T146 (inoculation with strain T146 plus 400 mM NaCl). Scale bar = 10 cm. (B) Plant height of wild‐type and *TaPIN1*‐RNAi wheat plants under different treatments from (A). (C) Fresh weight of wild‐type and *TaPIN1‐*RNAi wheat plants under different treatments from (A). (D) Malondialdehyde (MDA) content in wild‐type and *TaPIN1*‐RNAi wheat plants under different treatments from (A). (E) Peroxidase (POD) activity in wild‐type and *TaPIN1*‐RNAi wheat plants under different treatments from (A).(F) Number of differentially expressed genes (DEGs) (|log₂FC| > 1) in the comparison between NaCl+T146 and NaCl treatments in Fielder and *TaPIN1*‐RNAi lines. (G) Heatmap of expression levels (FPKM) of selected auxin‐related genes in Fielder and *TaPIN1*‐RNAi lines under NaCl and NaCl+T146 treatments. Data are presented as mean ± SD (*n* ≥ 15; enzyme activity assays were performed in triplicate). Comparisons among four groups were performed by one‐way ANOVA, followed by Tukey HSD test. Different letters (a–c) indicate significant differences between groups (*p* < 0.05). [Color figure can be viewed at wileyonlinelibrary.com]

To investigate whether the salt tolerance conferred by *B. cereus* T146 is mediated through the auxin transporter TaPIN1 in wheat, we conducted phenotypic and molecular analyses using *TaPIN1*‐RNAi knockdown and overexpression lines (Yao et al. 2021). Under salt stress, wild‐type wheat (Fielder) showed significant reductions in fresh weight and plant height, while T146 inoculation largely restored these growth parameters to near‐normal levels (Figure 6A–C). In contrast, in *TaPIN1*‐RNAi lines (RNAi #1 and #2), T146 inoculation exerted only a modest restorative effect on fresh weight and plant height under salt stress. Nevertheless, plants subjected to the NaCl combined with T146 treatment still exhibited a slight growth advantage compared with those treated with NaCl alone. (Figure 6A–C), indicating that loss of *TaPIN1* function renders wheat insensitive to T146's growth‐promoting effects. In *TaPIN1*‐overexpression lines (OE #3 and #4), growth under salt stress was similar to that of wild‐type plants, but T146 inoculation did not further enhance plant height or fresh weight (Figure S6), suggesting that elevated *TaPIN1* expression may partially mimic T146's effects or that the beneficial response has reached a ceiling.

Physiological analyses revealed that salt stress increased malondialdehyde (MDA) content and peroxidase (POD) activity in wild‐type plants, indicating oxidative damage. T146 inoculation significantly reduced these stress markers, confirming its role in mitigating salt‐induced harm (Figure 6D–E). In *TaPIN1*‐RNAi lines, T146 treatment only partially restored MDA and POD levels, consistent with the attenuated phenotypic response (Figure 6D–E).

Transcriptomic profiling revealed that T146 treatment (NaCl + T146 vs. NaCl) led to the identification of more than 6,000 differentially expressed genes (DEGs) in wild‐type plants, whereas fewer than 2,000 DEGs were detected in the *TaPIN1*‐RNAi background (Figure 6F). This indicates that *TaPIN1* is a critical mediator underlying the T146‐dependent modulation of gene expression under salt stress. Expression analysis of auxin‐related genes revealed that T146 strongly induced *TaPIN1*, *TaPIN4*, *TaAUX1*, *TaLAX2*, and several *SAUR* family genes in wild‐type plants (Figure 6G). This response was markedly reduced or absent in *TaPIN1‐*RNAi lines (Figure 6G), demonstrating that *TaPIN1* silencing disrupts auxin transport and signalling, thereby diminishing T146's beneficial effects. In summary, these results indicate that TaPIN1 contributes to T146‐enhanced salt tolerance in wheat. T146 treatment was associated with increased TaPIN1 expression, which may facilitate auxin transport and downstream signalling, correlating with improved antioxidant capacity and growth recovery under salt stress.

## 4 Discussion

Soil salinization poses a pervasive threat to global agricultural productivity. In this study, we show that the rhizobacterium *B. cereus* T146 enhances salt tolerance in wheat, with more pronounced effects observed under non‐sterilised soil conditions (Figure 1) (Xu et al. 2022). Integrating physiological, microbiological, molecular, and genetic analyses, our data support a model in which T146 acts through modulation of the host's auxin signalling pathway, with the auxin transporter PIN1 playing a central role in this response.

The observation that T146's beneficial effects were more pronounced in non‐sterilised soil suggests that its mode of action extends beyond a direct plant‐microbe interaction. The metagenomic sequencing data confirmed that T146 inoculation mitigates the salt‐induced reduction in microbial diversity and enriches beneficial taxa such as *Pseudomonadota*, *Alphaproteobacteria*, and nitrogen‐fixing genera like *Devosia* and *Mesorhizobium* (Figure 2) (Pan et al. 2022; Zhao et al. 2025). Furthermore, the functional prediction indicated an enhancement in metabolic pathways and stress‐responsive functions within the rhizosphere microbiome (Zheng et al. 2024). These results indicate that T146 inoculation is associated with distinct shifts in the rhizosphere microbial community structure, specifically the enrichment of potentially beneficial taxa that may contribute concurrently to enhanced plant growth under stress. Metagenomic analysis further revealed that T146 inoculation significantly reshaped the *Pseudomonas* population in the wheat rhizosphere under salt stress. Compared to the salt‐stressed control, the absolute abundance of *Pseudomonas* was markedly restored and stabilised by T146 treatment (Figure S8A), with high consistency across biological replicates. Consistently, culturomics showed that *Pseudomonas* became the dominant cultivable genus (37.04%) in the T146+NaCl rhizosphere (Figure S2B), indicating that the isolated strains represent true in situ enriched populations rather than opportunistic contaminants. To assess their functional relevance, we performed a stratified analysis of auxin biosynthesis genes (e.g., *amiE* [K00274], *nthA* [K00626], *aldH* [K01817]). Strikingly, the contribution of *Pseudomonas* to these genes was negligible under salt stress alone but increased significantly upon T146 inoculation (*p* < 0.05), recovering to or exceeding levels in non‐saline controls (Figure S8B). This provides direct evidence that T146 recruits specific *Pseudomonas* subpopulations that actively harbour the genetic potential for IAA synthesis, thereby contributing to the restoration of auxin homoeostasis in the host. The transcriptomic profiling of wheat roots offered crucial insights into the direct molecular interplay between T146 and the host plant. T146 treatment markedly attenuated the extensive transcriptional reprogramming triggered by salt stress. Notably, GO enrichment analysis revealed a significant involvement of auxin‐related processes, including transport, metabolism, and signalling (Figure 3) (Yu et al. 2022). This finding directed our investigation toward the auxin pathway. Further examination of specific gene expression showed that salt stress strongly suppressed key auxin biosynthetic genes (e.g., *YUC11*) and polar transport genes (e.g., *PIN1*, *PIN2*, *PIN3*) (Naramoto 2017; Xu et al. 2021), while T146 treatment effectively restored their expression (Figure 3). This transcriptional recovery was functionally consequential, as it was correlated with a measurable phenotypic rescue of lateral root development under salinity (Figure 4).

Subsequent validation experiments in wheat and *Arabidopsis* robustly supported this hypothesis. The rescue of salt‐inhibited root growth (primary root length, lateral root number, and root hair length) by T146, and most importantly, the complete abolition of this rescue by the auxin transport inhibitor NPA, unequivocally demonstrated that T146's beneficial effects are dependent on functional auxin transport (Figure 4) (Jiménez‐Vázquez et al. 2020). Moreover, in wheat, T146 significantly upregulated the expression of key regulatory genes involved in lateral root initiation, including *PLT3*, *PLT7*, *GLV6*, and *BDL* (Figure 4) (Du and Scheres 2017; Fernandez et al. 2015; Goh et al. 2012). This coordinated genetic response likely underpins the observed architectural improvements in the root system. The restoration of DR5::GFP expression (indicating auxin accumulation) in the root apex of T146‐treated, salt‐stressed plants provided direct visual evidence of T146's role in maintaining auxin homoeostasis under stress (Figure 5). This restoration was correlated with the specific upregulation of PIN1, PIN2 and PIN3 protein abundance (Figure 5), suggesting that T146 fine‐tunes polar auxin transport by modulating the expression of key auxin efflux carriers.

The most significant finding of this study is the identification of *TaPIN1* as a critical mediator of T146‐induced salt tolerance in wheat (Figure 6) (Bawa et al. 2022; Yao et al. 2021). The observation that *TaPIN1*‐RNAi lines failed to respond to T146 inoculation and showed no significant improvement in biomass, growth, or mitigation of oxidative damage under salt stress provides strong genetic evidence that *TaPIN1* functions as a central downstream target of T146 (Figure 6). The RNA‐seq data from these silenced lines were particularly revealing: the dramatic reduction in the number of differentially expressed genes in response to T146 in the *TaPIN1*‐RNAi background indicates that *TaPIN1* is not just one of many responsive genes but is a key hub through which T146 orchestrates a broad transcriptional response to salt stress. The dampened response of other auxin‐related genes (e.g., *TaPIN4*, *TaAUX1*, *TaLAX2*, and *SAURs*) in *TaPIN1*‐silenced lines suggests that T146 may enhance salt tolerance by upregulating *TaPIN1* expression, which in turn modulates auxin transport and downstream signalling pathways, ultimately improving antioxidant capacity and promoting growth recovery under stress (Bao et al. 2024; Guo et al. 2018). The partial effect in overexpressing lines implies a potential saturation of the response pathway.

To better validate how the recruited *Pseudomonas* strains contribute to enhanced salt tolerance, we specifically isolated the T146‐recruited *Pseudomonas* strain TN12 and evaluated its salt tolerance–promoting activity through independent inoculation assays in both wheat and *Arabidopsis*, as well as through co‐inoculation with T146 (Figure S7). Phenotypic analyses in both wheat and *Arabidopsis* reveal a cooperative interaction between T146 and the recruited *Pseudomonas* strain TN12 in enhancing plant salt tolerance. These observations are consistent with the interpretation that T146‐mediated rhizosphere microbiome remodelling may be functionally reinforced by specific *Pseudomonas* taxa, in line with previous reports (Zheng et al. 2024). Importantly, the enhanced performance of the T146–TN12 consortium suggests a cooperative interaction, potentially reflecting complementary activities that promote robust plant stress resilience.

Beyond efficcy, biosafety is a prerequisite for agricultural deployment, particularly in light of th‐ taxonomic placement of T146. Phylogenetic analysis (Figure S9) clearly separates T146 from toxin‐producing lineages and clusters it with non‐pathogenic PGPR such as *Bacillus cereus* AR156 (Bai et al. 2020; Ivanova et al. 2003; Jiang et al. 2017; Jiménez et al. 2013). For future applications, we propose rhizosphere‐targeted delivery strategies, including seed coating and root drenching, to spatially confine bacterial colonisation to the root zone and minimise contact with aboveground edible tissues, thereby reducing the risk of entry into the food chain. These approaches will be coupled with formulation optimisation and field plot trials, alongside systematic monitoring of environmental persistence and biosafety, including potential impacts on soil microbial communities and surveillance of virulence‐associated genes, to ensure the safety and stability of large‐scale application.

**Figure 7:**
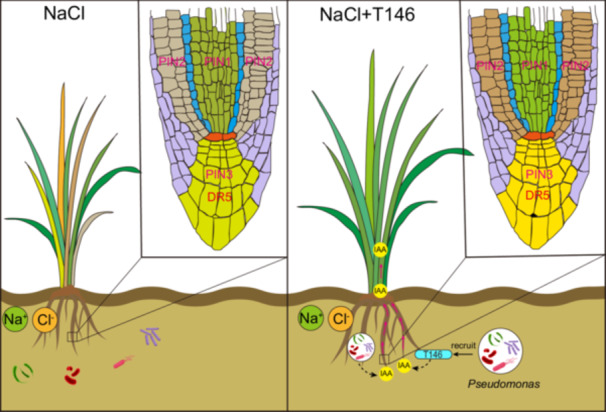
Working model: *B. cereus* T146 enhances plant salt tolerance by modulating the auxin signalling pathway and reshaping the rhizosphere microbiome. Under salt stress, plant growth is inhibited due to disrupted auxin homoeostasis and oxidative damage. Inoculation with strain T146 (right) ameliorates salt stress through a dual mechanism: (1) Indirect pathway (rhizosphere microbiome): T146 recruits beneficial IAA‐producing *Pseudomonas* species in the rhizosphere, increasing the local auxin pool. (2) Direct pathway (plant auxin signalling): T146 and/or the microbially‐derived IAA directly activate the host's auxin signalling pathway. This leads to the upregulation of *PIN1*, *PIN2*, and *PIN3* protein expression, enhancing polar auxin transport. Consequently, auxin accumulation (reflected by DR5 activity) in the root tip is restored, promoting root system architecture remodelling and improving stress resilience. The solid arrows represent processes supported by direct experimental evidence from this study. [Color figure can be viewed at wileyonlinelibrary.com]

Integrating the findings presented here, we propose a dual‐mechanism model for *B. cereus* T146 action (Figure 7). T146 inoculation coincides with the modulation of the host auxin signalling network, characterised by the sustained expression of key PIN auxin transporters, particularly PIN1 and PIN2. These molecular changes likely facilitate polar auxin transport and support root auxin homoeostasis, enabling adaptive remodelling of root system architecture under salt stress (Jiménez‐Vázquez et al. 2020). Parallel to these host responses, T146 colonisation is associated with distinct shifts in the rhizosphere microbiome, specifically the enrichment of functionally active taxa such as IAA‐producing Pseudomonas, which may offer concurrent contributions to growth promotion and stress mitigation (Yuan et al. 2023). Collectively, our work provides functional and genetic evidence demonstrating an association between a plant growth‐promoting rhizobacterium, a specific host auxin transporter pathway, and enhanced salinity tolerance. These findings underscore the potential of microbe‐based strategies that target conserved phytohormonal networks to sustainably improve crop performance in salt‐affected agricultural systems.

## Conflicts of Interest

The authors declare no conflicts of interest.

## Supporting information

Supporting File 1

Supporting File 2

## Data Availability

The original datasets generated in this study are publicly available in the NCBI repository: the soil metagenomic data and wheat transcriptomic data are deposited under the same BioProject accession number PRJNA1347134, and can be directly downloaded from the NCBI website (https://www.ncbi.nlm.nih.gov/sra). All other raw data supporting the findings of this study are available from the corresponding author upon reasonable request.
