# Supplementary material for: Bacillus cereus T146 Enhances Wheat Salt Tolerance by Restructuring the Rhizosphere Microbiome and Activating TaPIN1‐Dependent Auxin Transport
Source: Plant Cell Environ. 2026 Apr 28;49(8):5703–19. doi: 10.1111/pce.70567 (PMC13353569; doi:10.1111/pce.70567)
Supplement: Supplementary file 1 — Supporting File 1 [file PCE-49-5703-s001.pdf]

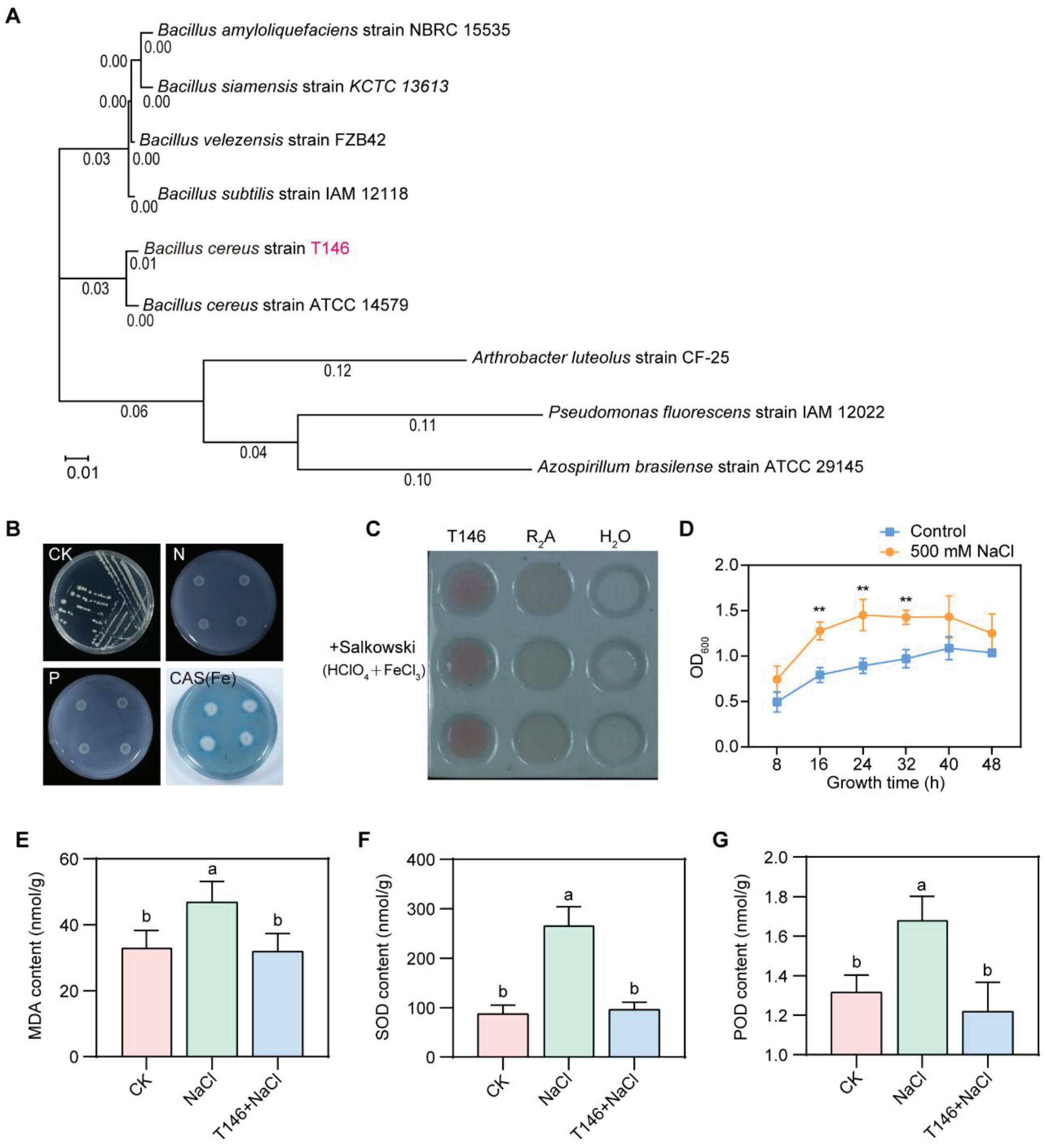


**Figure. S1 Plant growth-promoting traits of *Bacillus cereus* T146 and its mitigation of oxidative damage in wheat under salt stress. (A)** Maximum likelihood phylogenetic tree based on 16S rRNA gene sequences, showing the evolutionary position of strain T146 among related bacterial species. Bootstrap values (based on 1000 replicates) are shown at the nodes. The scale bar represents 0.01 substitutions per nucleotide position. **(B)** Plant-growth-promoting functional traits of T146, including phosphorus (P) solubilization, nitrogen (N) fixation and siderophore production [CAS(Fe) assay]. **(C)** Qualitative assay results confirming the ability of strain T146 to produce indole-3-acetic acid (IAA). **(D)** Growth curve of T146 cultured in liquid medium with or without NaCl stress, demonstrating its tolerance to salinity. **(E)** Malondialdehyde (MDA) content determination in Fielder wheat under different treatments in non-sterilized soil. **(F)** Superoxide dismutase (SOD) activity determination in Fielder wheat under different treatments in non-sterilized soil. **(G)** Peroxidase (POD) activity determination

in Fielder wheat under different treatments in non-sterilized soil. In (E), (F), and (G), data are presented as mean ± SD ($n = 3$ independent biological replicates). Different lowercase letters above bars indicate significant differences among treatments within the same soil condition ($P < 0.05$, Student's *t*-test).

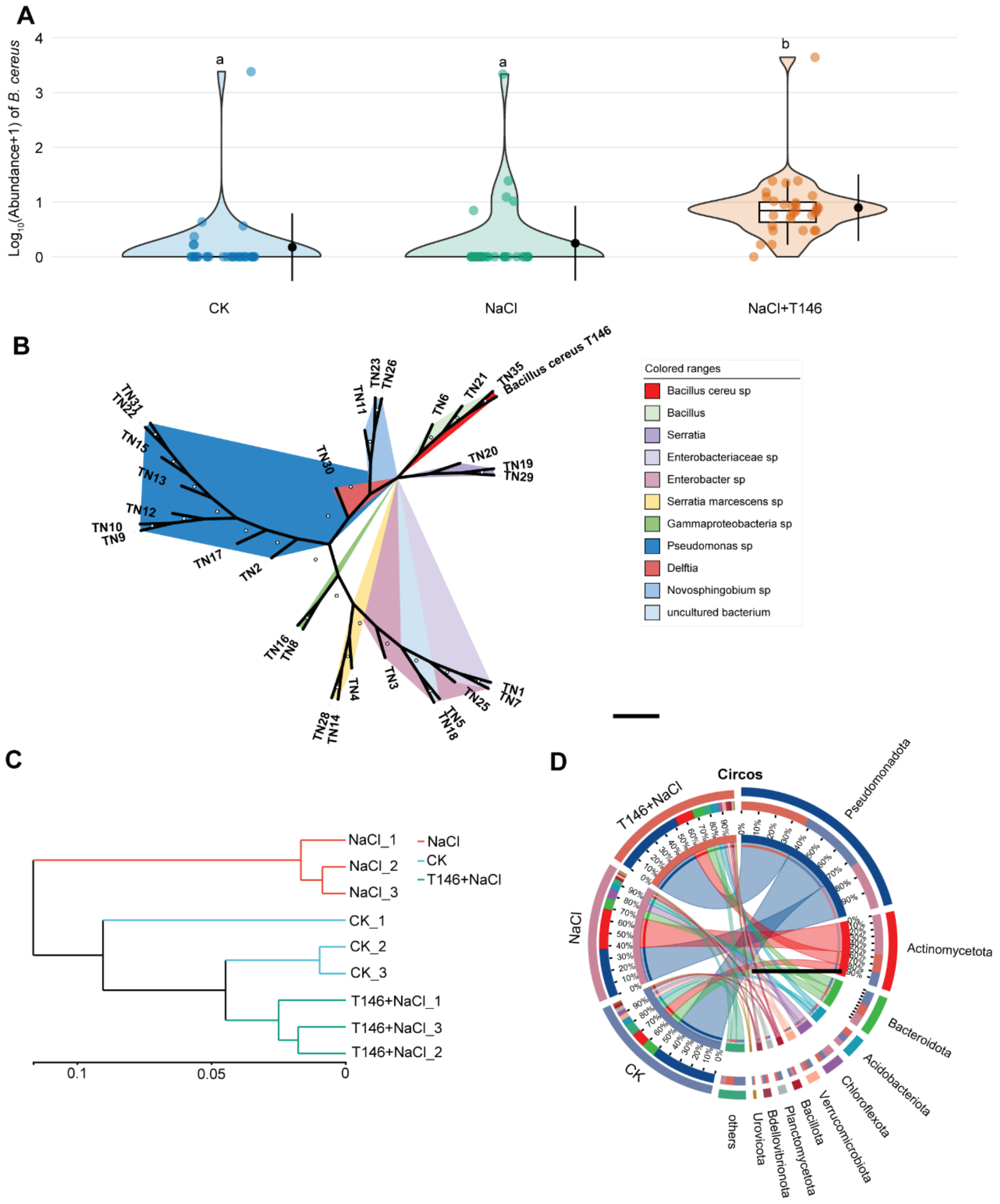


**Figure. S2 Supplementary analysis of wheat rhizosphere microbial community composition and colonization of *B. cereus* T146 in the wheat rhizosphere under salt stress.** (**A**) Effects of Different Treatments on *B. cereus* Abundance, with three treatments including control (CK), salt stress (NaCl), and salt stress combined with T146 inoculation (NaCl+T146). (**B**) Phylogenetic tree based on 16S rRNA gene sequences showing the relationship between the inoculated strain T146 and the isolated rhizosphere strain TN35. Bootstrap values (1000 replicates) are shown at branch nodes. The scale bar represents 0.01 substitutions per nucleotide position. **(C)** Hierarchical clustering tree (NR dendrogram) of samples based on microbial community composition, showing similarity among biological

replicates. **(D)** Circos plot illustrating the abundance relationships of major microbial phyla across different treatment groups at the phylum level. Treatment conditions: CK (control), NaCl (400 mM NaCl), T146+NaCl (inoculation with *B. cereus* T146 + 400 mM NaCl). In (**A**), data are presented as mean ± SD ($n$ = 3 independent biological replicates). Violin plots show data distribution, boxplots show median and IQR. Different lowercase letters above the columns indicate significant differences among treatments ($P < 0.05$, Student's $t$-test).

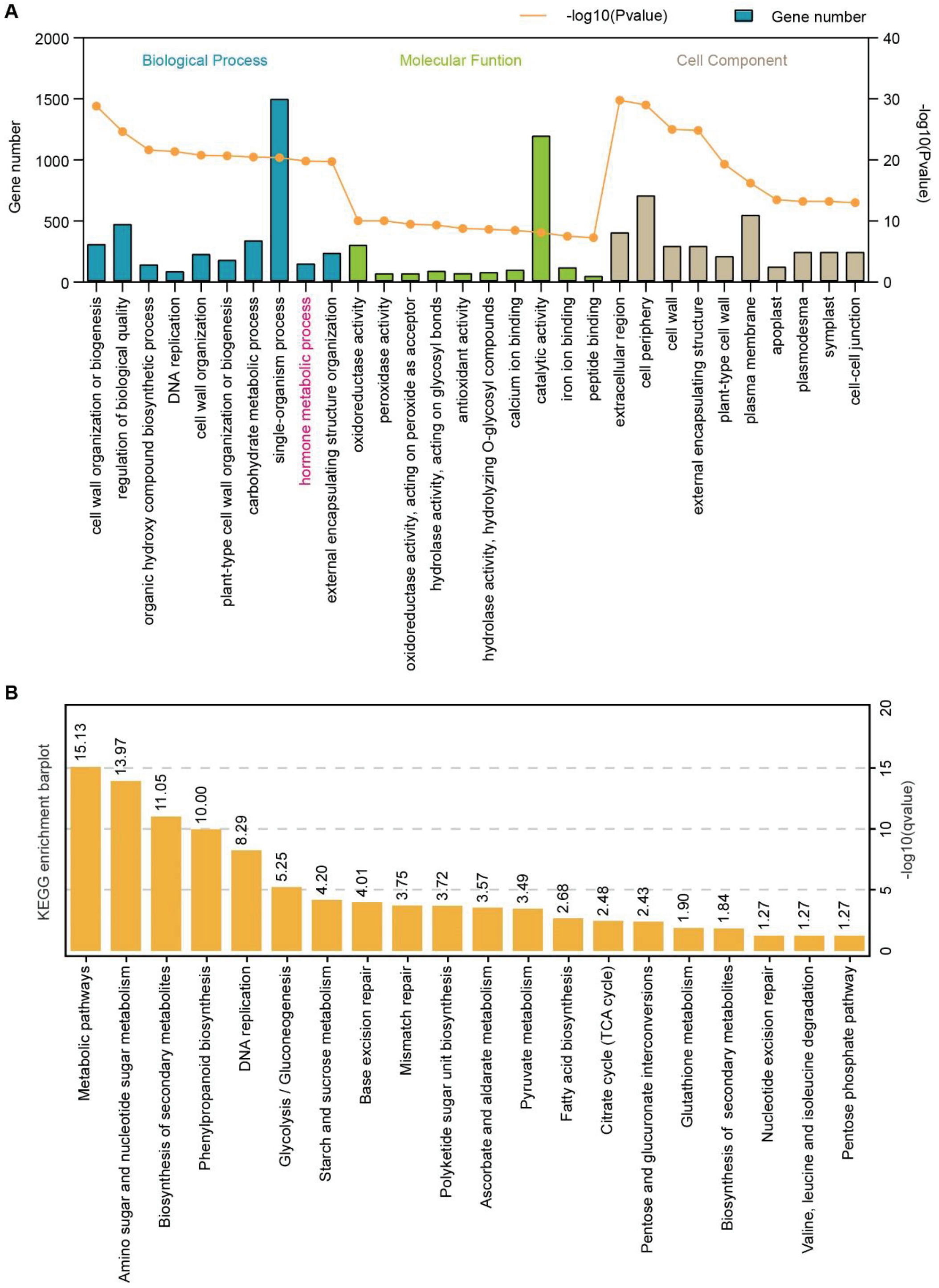


**Figure. S3 GO term enrichment and KEGG pathway analysis of differentially expressed genes (DEGs) between NaCl and T146+NaCl treatments. (A)** Gene Ontology (GO) enrichment analysis for all DEGs specifically identified from the NaCl+T146/NaCl comparison. The bar plot shows representative significantly enriched GO terms (from the categories Biological Process, Molecular Function and Cellular Components) among the DEGs. **(B)** KEGG pathway enrichment analysis for all DEGs identified from the NaCl+T146/NaCl

comparison. The bar plot shows the top significantly enriched KEGG pathways. The X-axis represents the enrichment factor (GeneRatio), and the Y-axis lists the enriched pathways.

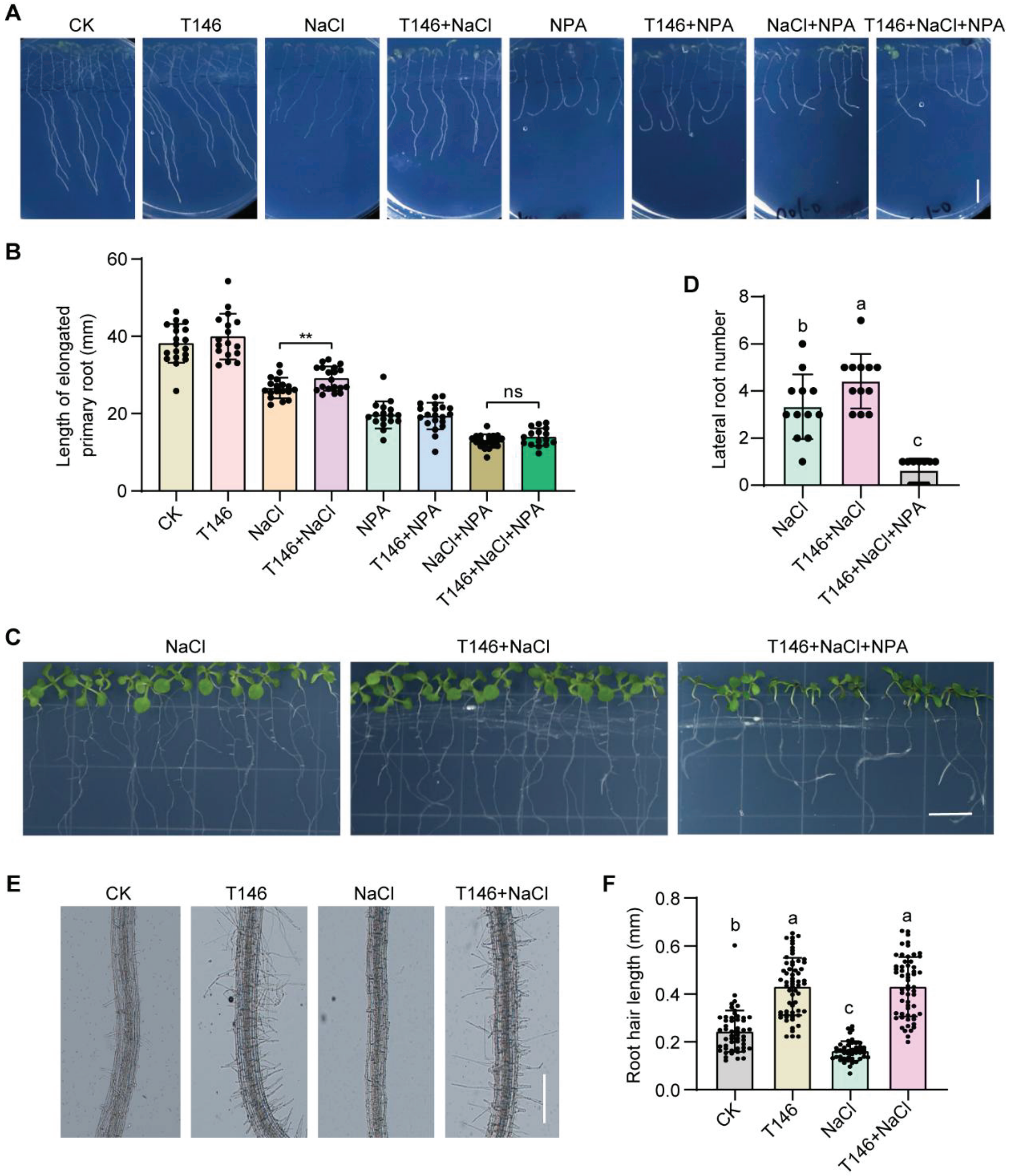


**Figure. S4 The auxin transport inhibitor NPA specifically blocks the restorative effect of T146 on salt-inhibited root growth. (A)** Representative images of *Arabidopsis* seedlings under various treatments, highlighting the comparison between NaCl+NPA and T146+NaCl+NPA. 4-day-old seedlings were transferred and grown vertically for 5 days under the following treatments: CK (control), T146 (inoculation with *B. cereus* T146), NaCl (100 mM NaCl), T146+NaCl (T146 + 100 mM NaCl), NPA (10 μM NPA), T146+NPA (T146 + 10 μM NPA), NaCl+NPA (100 mM NaCl + 10 μM NPA), T146+NaCl+NPA (T146 + 100 mM NaCl + 10 μM NPA). Scale bar = 1 cm. **(B)** Quantitative analysis of elongated primary root length from (A). Data are presented as mean ± SD ($n \geq 10$). ns, not significant, $^{**}P < 0.01$, Student's $t$-test.

**(C)** NPA treatment abolishes the T146-induced growth promotion of lateral roots under salt stress. Scale bar = 1 cm. **(D)** Quantitative analysis of lateral root number from (C). Data are presented as mean ± SD ($n \geqslant 10$). Different lowercase letters (a, b, c) above the columns indicate significant differences among treatments. For multi-group comparisons, one-way ANOVA followed by Tukey's HSD post-hoc test was applied. **(E)** Root hair phenotype of seedlings under different treatments (CK, T146, NaCl, T146+NaCl). 4-day-old wild-type seedlings were transferred to 1/2 MS solid media and grown vertically for 7 days. Scale bar = 1 mm. **(F)** Quantification of root hair length from (E). Data are presented as mean ± SD ($n \geqslant 10$). Different lowercase letters (a, b, c) above the columns indicate significant differences among treatments. For multi-group comparisons, one-way ANOVA followed by Tukey's HSD post-hoc test was applied.

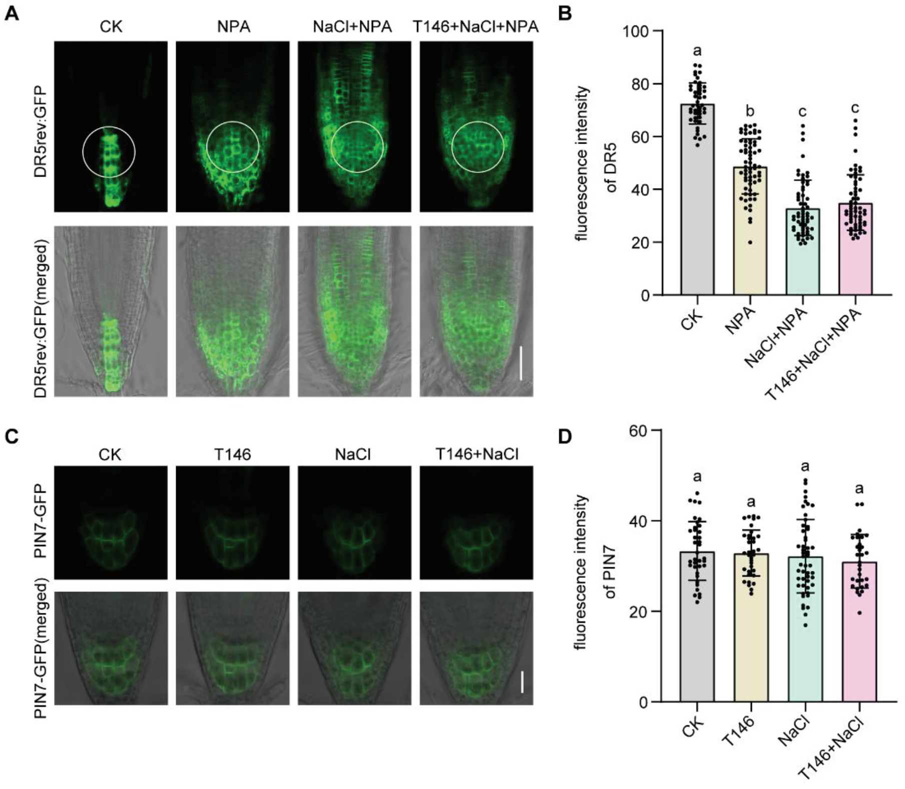


**Figure. S5 The expression and localization of PIN7 are not affected by T146 inoculation or salt stress. (A)** Representative confocal microscopy images of root tips expressing *DR5 rev:GFP* under different treatments (CK, NPA, NaCl+NPA, T146+NaCl+NPA). Bright-field and merged channels are shown. Four-day-old seedlings were transferred and grown on fresh medium for 5 days before imaging. Scale bar = 50 μm. **(B)** Quantification of *DR5 rev:GFP* fluorescence intensity in the root tip cells marked by circle lines from (A). **(C)** Representative confocal microscopy images of *Arabidopsis* root tips expressing *PIN7-GFP* under different treatments: CK (control), T146 (inoculation with *B. cereus* T146), NaCl (100 mM NaCl), T146+NaCl (T146 + 100 mM NaCl), NPA (10 μM NPA), and T146+NaCl+NPA (T146 + 100 mM NaCl + 10 μM NPA). Bright-field and merged channels are shown. Scale bar = 20 μm. **(D)** Quantitative analysis of PIN7-GFP fluorescence intensity in root tips. Data are presented as mean ± SD ($n \geqslant 10$ roots). Different lowercase letters (a, b, c) above the columns indicate significant differences among treatments. For multi-group comparisons, one-way ANOVA followed by Tukey's HSD post-hoc test was applied ($P < 0.05$).

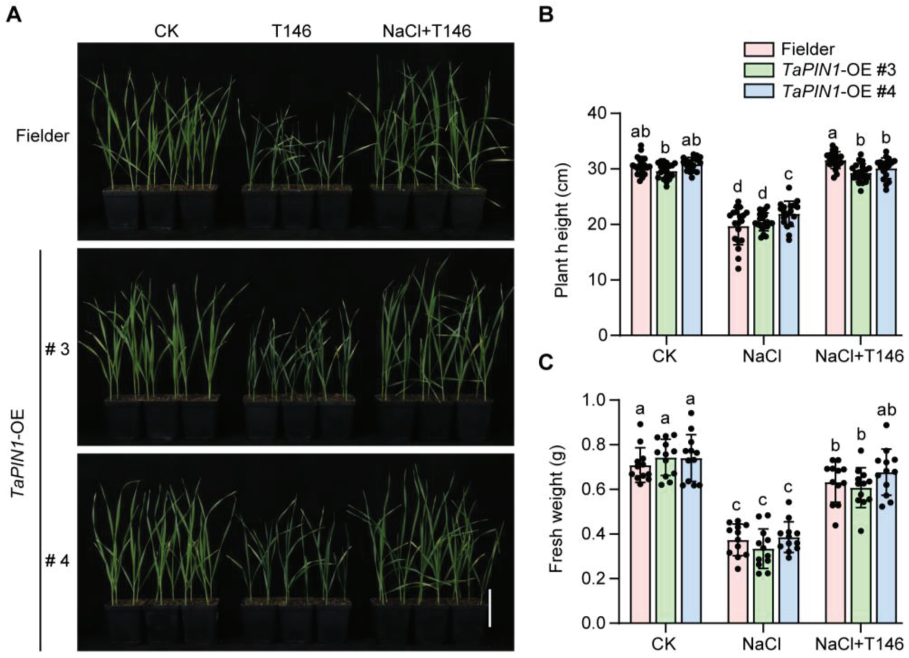


**Figure. S6 Phenotypic analysis of *TaPIN1* overexpression lines in response to salt stress and T146 inoculation. (A)** Representative images of wild-type (Fielder) and *TaPIN1*-overexpression (OE) wheat plants (independent lines #3 and #4) under different treatments: CK (control), NaCl (400 mM NaCl), and T146+NaCl (inoculation with *B. cereus* T146 + 400 mM NaCl). Scale bar = 10 cm. **(B)** Plant height of wild-type and *TaPIN1*-OE wheat plants under different treatments from (A). **(C)** Fresh weight of wild-type and *TaPIN1*-OE wheat plants under different treatments from (A). Data are presented as mean ± SD ($n \geq 12$ plants). Different lowercase letters (a, b, c) above the columns indicate significant differences among treatments. For multi-group comparisons, one-way ANOVA followed by Tukey's HSD post-hoc test was applied ($P < 0.05$).

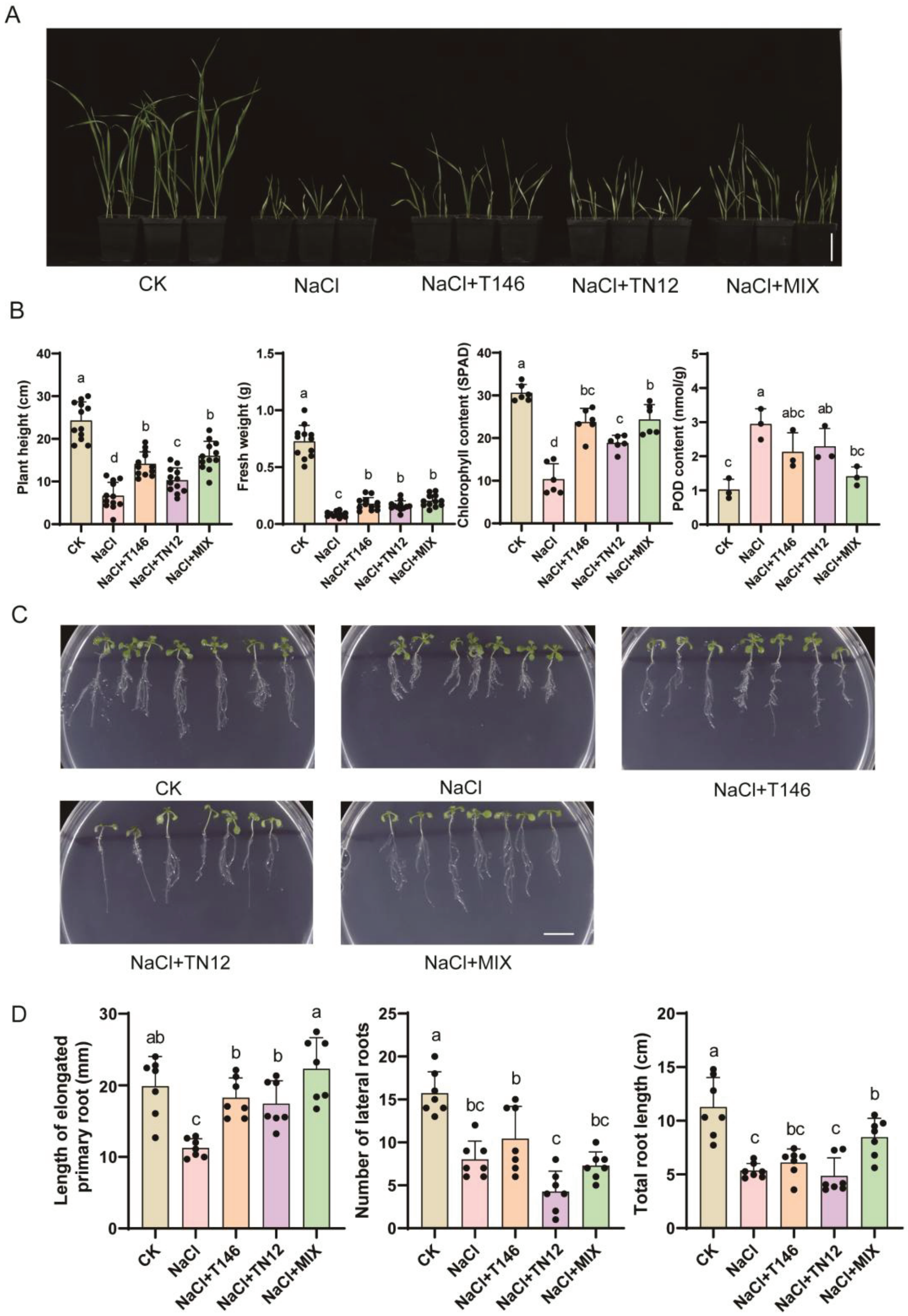


**Figure. S7 Phenotypic analysis of wheat and *Arabidopsis* in response to salt stress under single-strain (T146, TN12) and mixed consortium (MIX) inoculation. (A)** Representative images of wild-type (Fielder) wheat under different treatments: CK (control), NaCl (400 mM NaCl), NaCl+T146 (inoculation with strain T146 + 400 mM NaCl), NaCl+TN12 (inoculation with strain TN12 + 400 mM NaCl), and NaCl+MIX (co-inoculation with strain T146 + strain TN12 + 400 mM NaCl). Scale bar = 10 cm **(B)** Plant height, Fresh weight, Chlorophyll content (SPAD value) and POD content of wheat plants under different treatments from (A). **(C)**

Representative images of *Arabidopsis* thaliana (Col-0) under different treatments: CK (control), NaCl (50 mM NaCl), NaCl+T146 (inoculation with strain T146 + 50 mM NaCl), NaCl+TN12 (inoculation with strain TN12 + 50 mM NaCl), and NaCl+MIX (co-inoculation with strain T146 + strain TN12 + 50 mM NaCl) Scale bar = 1 cm **(D)** *Arabidopsis* root traits: Length of elongated primary root and number of lateral roots. *Arabidopsis* root growth parameters: Total root length (Data sources: Figure S7C). Data are presented as mean ± SD ($n \geqslant 6$). Different lowercase letters (a, b, c) above the columns indicate significant differences among treatments. For multi-group comparisons, one-way ANOVA followed by Tukey's HSD post-hoc test was applied ($P < 0.05$).

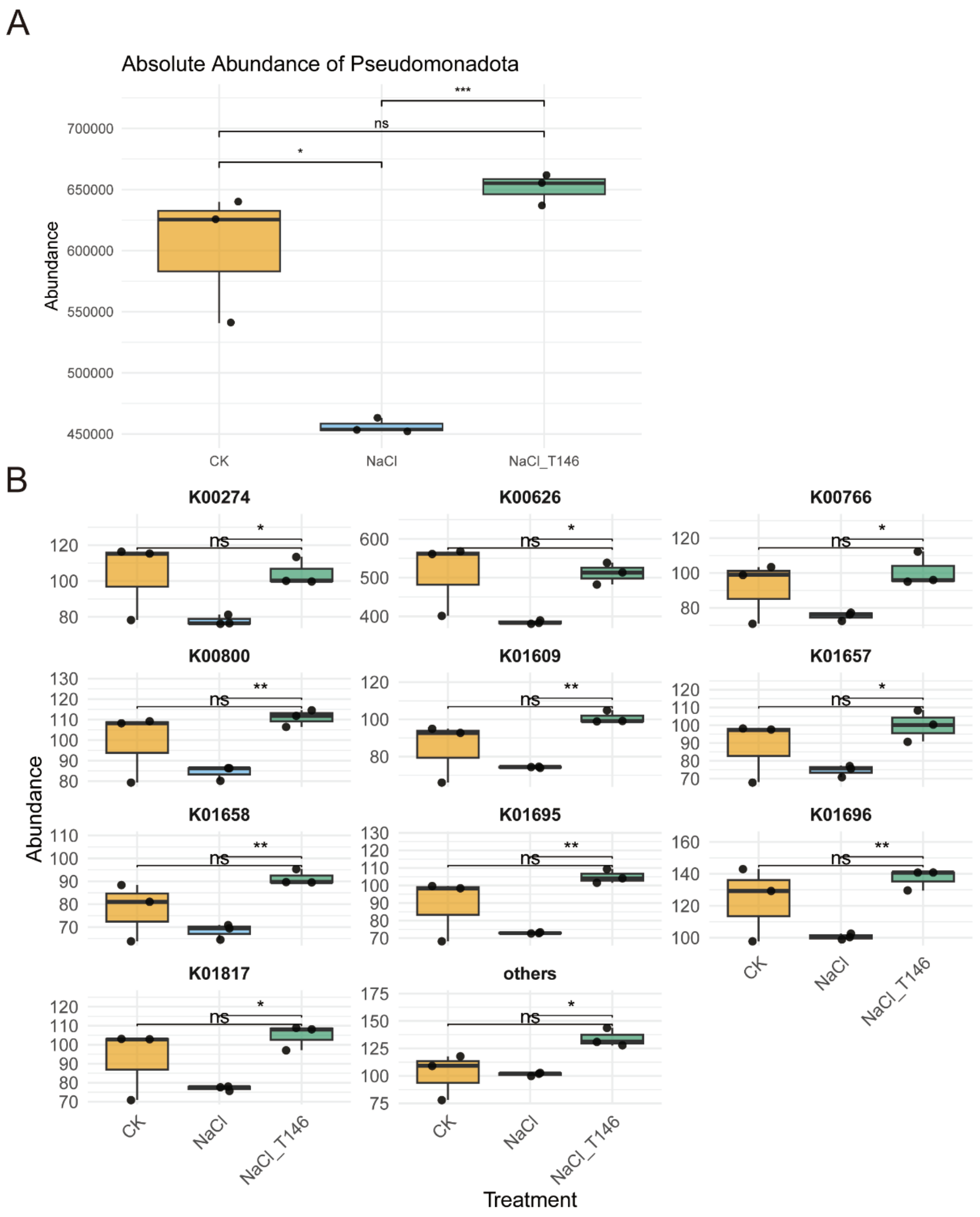


**Figure. S8 Absolute abundance of *Pseudomonadota* and the relative abundance of key functional genes in different treatments. (A)** Absolute abundance of *Pseudomonadot*a in CK, NaCl and NaCl-T146 treatments; **(B)** Relative abundance of key functional genes in CK, NaCl and NaCl-T146 treatments. Data are presented as mean ± SD ($n \geq 3$). ns, not significant, *$P <$ 0.05, **$P < 0.01$, ***$P < 0.001$ Student's $t$-test.

A

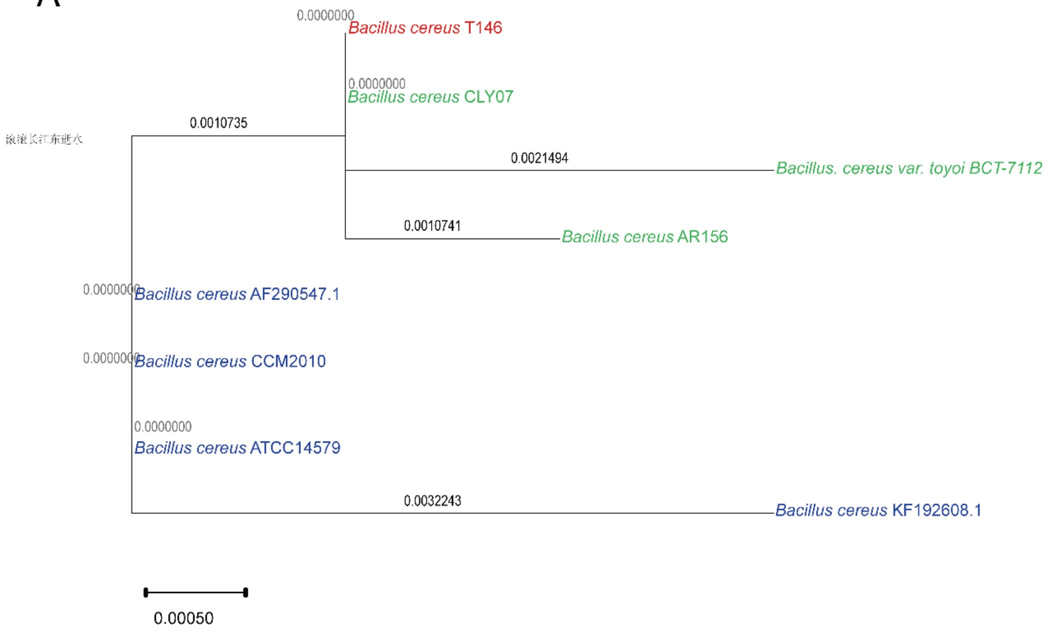


**Figure. S9 Phylogenetic tree of *Bacillus cereus* T146 and toxin-producing and non-pathogenic *Bacillus* strains. (A)** T146 is phylogenetically distinct from the toxin producing lineages and clusters closely with plant beneficial *B. cereus* clade. T146 is highlighted in red. Representative toxin producing strains (*B. cereus* AF290547.1, CCM2010, ATCC14579, KF192608.1) are indicated in blue, and non-pathogenic strains (*B. cereus* AR156, *B. cereus* CLY07, *B. cereus var. toyoi* BCT 7112) are shown in green. The branch values denote the evolutionary distances among the strains, with a scale bar of 0.0005 representing a nucleotide sequence evolutionary divergence of 0.0005 substitutions per site.
